# Experimental performance verification of an intelligent detection and assessment scheme for disturbances and imbalances of three-phase synchronous machine output using coherence estimators

**DOI:** 10.1038/s41598-024-76343-8

**Published:** 2024-11-01

**Authors:** R. A. Mahmoud

**Affiliations:** Misr University for Science and Technology (MUST), College of Engineering Science & Technology, Department of Electrical Power and Machines Engineering (PME), 6Th of October City, Giza, Egypt

**Keywords:** Synchronous machines, Motor-generator set, Series and shunt faults, Imbalance and disturbance, Coherence estimator, LABVIEW, Data Acquisition Card (DAC), Energy science and technology, Engineering

## Abstract

In this article, power quality disturbances, such as voltage and current unbalances, series and shunt faults, are monitored in three-phase synchronous machines. Because the imbalances affect machine efficiency and service life, it is essential to figure out whether the three-phase voltages/currents are balanced or unbalanced. An integrated detection algorithm is proposed to detect the unbalanced voltages/currents and various abnormal conditions precisely and quickly, which is based on the coherence estimator. Most existing detection methods require more than one cycle time to determine the contingency conditions, while the proposed technique detects these situations in a fast and discrete protection system. The suggested scheme acquires instantaneous measurements of the three-phase voltages and currents taken at the synchronous machine terminals in order to calculate fifteen coherence coefficients that are utilized to detect and assess any unbalance or trouble accurately and swiftly. A new proposal for imbalance and disturbance indicators based on the coherence estimators is developed in this study. Multiple test cases have been conducted to validate the proposed algorithm capabilities using a real power system, which includes a motor-generator set, a three-phase load and measurement transformers. The experimental results have been revealed that the relay reliability and accuracy percentages have been found to be 98.28% and 98.52%, respectively. The quantitative findings of the imbalance and disturbance assessment are recorded.

**Supplementary Information:**

The online version contains supplementary material available at 10.1038/s41598-024-76343-8.

## Introduction

Different challenging problems such as voltage and current imbalances and shunt faults may pose a threat to synchronous machines, which are one of the important components of electrical power networks^1–3^. The imbalance and fault incidents usually lead to troubles in the power quality parameters of the electrical quantities^4,5^. In fact, the main reasons of the unbalanced voltages/currents are as follows: (1) series and shunt faults^6^, (2) circuit breaker poles discrepancy^7^, (3) asymmetry in network topology^8^, (4) unsymmetrical transformer windings^9,10^, and (5) the uneven distribution between the three-phase loads^11^. Subsequently, the three-phase apparent powers transferred between the generator and the load are unequal, causing unbalanced voltages and currents at the load end^12^. The voltage and current imbalances result in the following problems: (1) excessive power losses, (2) heating up the electrical equipment, (3) reduction in the efficiency of rotating machines, transformers, cables, or lines, (4) weakening the resultant of machine torque, and (5) the possibility of system instability/loss-of-synchronism^13,14^. In addition, the unbalanced conditions of power converters originate characteristic and uncharacteristic harmonics^15^.

Several techniques were presented for monitoring and evaluating the voltages and currents imbalance, as well as series and shunt faults. Changing the system configuration through the feeder switching operations may regulate the loads distribution, which is based on allocation of single-phase loads equally across the three-phase system^16^. Static Volt-ampere-reactor Compensation (SVC) in the power grid provides many advantages such as a voltage regulation, load balance, and system stability improvement^17^. In^18^, the voltage imbalance identification was dependent on quantifying the sum (Vsum) and the space vector (Vspace) of the three load voltages at each sample; this is to accelerate the detection time of the voltage imbalance. It was found that most of the AI methods, used in machines fault diagnosis including ANN, have low precision in fault diagnosis because of the restriction of over-fitting and long convergence time^19^. In^20^, Nuisance Attribute Projection (NAP) algorithm was used to enhance the maximum fault classification accuracy for inter-turn faults applied to a small SG model. A voltage-controlled time overcurrent relay, which is a backup protection of differential relay, was proposed in^21^. A statistical approach in^22^ was presented for short-circuit current detection, fault type classification and fault location identification using deep convolutional transfer learning network. The paper^23^ proposed an analytical approach to identify, classify, and locate the internal phase-to-ground faults and inter-turn faults in the SG stator windings using harmonic components of the three-phase voltages. In^23^, Decision Tree (DT) was processed to extract the third and the fourth harmonic components of the voltage waves and the fundamental component of the residual voltage, which were used to find out the inter-turn faults and the number of the shorted turns, besides the faulty phase to-ground is detectable. The article^24^ developed an analytical method based on the coherence concept and Lagrangian function to isolate the generator subjected to an out-of-step incident, and to appreciate the system instability. The technique in^25^ was based on an alienation function-based numerical method for sensing three-phase currents imbalance of synchronous machine stator windings. The algorithm in^26^ relied on Pearson correlation and a sensitivity booster for detecting different fault situations, which was verified on the experimental model of the motor-generator set. The paper^27^ presented a scheme of voltage disturbance identification, evaluation, and classification using conjugate gradient back-propagation based Artificial Neural Network (ANN). A Wavelet Transform (WT)-based Power Quality Disturbances (PQDs) detection approach for multiple signals in a real-time environment was proposed in paper^28^. The method in^29^ was dependent on a fuzzy-based technique using cross-correlation function for detecting and classifying single and combined PQDs; where, the cross-correlation was applied as feature extraction tool to reduce the impact of random ripples inserted in the signal as well as low computational burden; while, a fuzzy classifier tool was used for classifying faults in real-time system. A Fractional Fourier Transform (FRFT) based-classification model for PQDs was used in the paper^30^. The paper^31^ introduced an advanced Digital Signal Processing (DSP)-based method using symmetrical components and Phase-Locked Loop (PLL). Several solutions based on the analysis of vibrations on rotating shafts were described in^32–34^ to find out imbalance or if roller bearings were damaged. In three-phase systems, unbalance assessment methods were used in^35,36^, and power quality evaluation was presented in^37^. Multiple methods demonstrated a reasonable solution for identifying turn-to‐turn faults^38,39^, which are a critical issue for some protection systems of the AC machine windings.

Some existing protection methods have the following imperfections:

1. The operating speed of the numerous relays is relatively late because they are based on the RMS values of the voltage and current curves^35,37,40–43^,
2. Some methods have operating/tripping-characteristics that are open^21,30,31^,
3. Some protection criterion is more complicated than others^20,23,24^,
4. A variety of mathematical formulas of various sub-algorithms are used to perform multi-function digital protection^24,29,31,37^,
5. Some relays need different threshold values for different parameters, such as voltage amplitude, current amplitude, phase angle shift, frequency and time delay^21,31,37^,
6. Certain techniques provide limited redundant protection functions^18,25^.
7. Several protection methods have low safety, reliability, and accuracy for detecting fault/imbalance events^32–34^,
8. Low-pass filters may be needed for some algorithms because their analog input signals are susceptible to harmonics^21,30^,
9. Some methods are characterized by a low sensitivity, and are unable to modify their settings of the tripping-characteristics^32–34^,
10. Some protection schemes have unacceptable accuracy in fault/imbalance detection and appreciation^19,27^, and
11. In some conventional methods, the di-symmetry coefficient is used to assess the unbalance degree by estimating the ratio of the negative phase sequence component to the positive phase sequence component. Therefore, these approaches ignore the presence of the zero phase sequence component^3,6,8,9,16–18,36^.

This work proposes an integrated detection and appreciation scheme for disturbances and imbalances of three-phase voltages and currents measured at the terminals of the synchronous machine stator windings using coherence estimators. The proposed protection uses fifteen coherence estimators, which are calculated for the six electrical signals, to detect and assess any contingency and treat the above problems related to the conventional digital relays. A new proposal to measure the intensity level of imbalance and disturbance of the electrical signals is described. This paper presents an experimental verification and performance analysis of the intelligent detection and assessment scheme. This scheme represents a second defense line for machine primary protections.

For more clarification, there are differences between the currently submitted work and the previously published paper^43^. The former is based on the MSC (Mean Squared Coherence) coefficients of the voltage and current signals measured at a SG output, while the later was dependent on the alienation coefficients derived from the MSC coefficients. Therefore, the new paper applies tripping-characteristic curves based on the MSC estimators; whereas, the old paper was used tripping-characteristic curves based on the alienation estimators for the SG protection. Hence, the present work has taken more advantage of the results obtained by the old one^43^, where the response speed of the MSC-based algorithm is faster than the alienation-based algorithm. Moreover, the accuracy, security, dependability, and reliability percentages of the proposed protection can be improved. Furthermore, in this study, new proposed indicators for measuring the severity levels of the disturbance and di-symmetry for three-phase signals are deduced using the MSC coefficients.

The article is organized as follows: Section "Integrated protection algorithm" explains the proposed detection and appreciation algorithm for unbalance and disturbance events, and suggests new tripping-characteristics using the coherence estimators. Section "Power system model under test" introduces the three-phase electric power model under test and specifies the parameters of its components. The experimental verification and analysis of the suggested scheme are discussed in Section "Results and discussions". Section "Overall technique evaluation" provides the evaluation of the technique performance with its main strengths, and contributions to knowledge are listed in Section "Prominent contributions". Finally, conclusions are acquainted in Section "Conclusions".

## Integrated protection algorithm

### Coherence estimator

The coherence estimator is a computational method that measures the degree of linearity of any two variables (or data sets) by testing for similar frequency components. It is occasionally referred to as Magnitude-Squared Coherence, whose numerical value is between 0.0 and + 1.0^43–46^. If the relationship between the two variables is perfectly linear at a given frequency, the coherence magnitude is + 1.0. Whereas, the coherence factor will be 0.0 if their association is completely nonlinear. Therefore, the coherence estimator can be used to design a digital relay algorithm, which is able to find out abnormal/imbalance conditions of three-phase voltages and currents measured at the load terminals of the SG stator windings. In other sense, the estimator is capable of distinguishing between healthy and faulty sections of power systems. Besides, the intensity of the fault and imbalance events can be measured using the coherence. The coherence-based algorithm provides a redundant protection for the SG stator windings, which triggers appropriate circuit breakers only if the primary protection fails to find the fault situation or fails to open the SG circuit breakers. The coherence estimator has the following characteristics:

1. The coherence estimator, which is a real value bounded from + 1.0 to 0.0, allows authors to design a novel group of relay tripping-characteristics based on the coherence^44^,
2. The auto-coherence index describes the relation of the same electrical signal at two different time intervals. Whereas, the cross-coherence index depicts the relationship between two distinct electrical signals at the same time span,
3. In the situation of the auto-coherence value is close to 0.0, the relationship between the two data sets of the same electrical signal is weak and non-linear, while, the relationship between the two data sets of the same electrical signal is strong and linear when the auto-coherence value is close to + 1.0.

Similarly, when the cross-coherence value is situated outside of the range of + 0.25 ± *Δ*, the relationship between the two electrical signals (with the same measurement units) is non-linear. Whereas, the relationship between the two electrical signals is linear when the cross-coherence value is approaching + 0.25. Where, *Δ* is a selected small deviation.

4. A swap of two electrical variables does not affect the coherence index,
5. When the magnitude, frequency, symmetry, and shape of electrical signal(s) are stationary, the computed coherence index are also constant. Any presence of disturbance/imbalance will transfigure the coherence index.
6. When varying any electrical variable around its average value, the coherence index can be quantified using the deviation from this average,
7. The coherence index is an invariant scale and a pure value that does not change with measurement units, and
8. The coherence estimator is stable when the two electrical variables are balanced and normal. Whereas, it is unstable when the two electrical variables are unbalanced and abnormal.

The mathematical equations for computing the fifteen coherence estimators are described below:

#### Cross-coherence estimator for each two phase signals

The cross-coherence estimator (*Cg*_*sx*_) can be computed between each two-phase signals (*g*_*s*_*(n)* and* g*_*x*_*(n)*) of the two phases *‘S’* and* ‘X’,* respectively, as shown below^43–46^:

1 $$Cg_{sx} (k) = \frac{{\left[ {} \right.(\begin{array}{*{20}c} {} \\ {} \\ \end{array} \sum\limits_{n = 0}^{N - 1} {g_{s1} (k) \times g_{x1} (k)} + g_{s2} (k) \times g_{x2} (k)\begin{array}{*{20}c} {} \\ {} \\ \end{array} )^{2} + \begin{array}{*{20}c} {} \\ {} \\ \end{array} (\begin{array}{*{20}c} {} \\ {} \\ \end{array} \sum\limits_{n = 0}^{N - 1} {g_{s1} } (k) \times g_{x2} (k) - g_{s2} (k) \times g_{x1} (k)\begin{array}{*{20}c} {} \\ {} \\ \end{array} )^{2} \left. {} \right]}}{{\sum\limits_{n = 0}^{N - 1} {} \left[ {} \right.(\begin{array}{*{20}c} {} \\ {} \\ \end{array} g_{s1} (k)\begin{array}{*{20}c} {} \\ {} \\ \end{array} )^{2} + (\begin{array}{*{20}c} {} \\ {} \\ \end{array} g_{s2} (k)\begin{array}{*{20}c} {} \\ {} \\ \end{array} )^{2} \left. {} \right] \times \sum\limits_{n = 0}^{N - 1} {} \left[ {} \right.(\begin{array}{*{20}c} {} \\ {} \\ \end{array} g_{x1} (k)\begin{array}{*{20}c} {} \\ {} \\ \end{array} )^{2} + (\begin{array}{*{20}c} {} \\ {} \\ \end{array} g_{x2} (k)\begin{array}{*{20}c} {} \\ {} \\ \end{array} )^{2} \left. {} \right]}}\begin{array}{*{20}c} {} & {} & {} \\ \end{array}$$

where,

$$g_{s1} (k) = \begin{array}{*{20}c} {\begin{array}{*{20}c} {} \\ {} \\ \end{array} \sum\limits_{n = 0}^{N - 1} {[g_{s} (n)\begin{array}{*{20}c} {} \\ {} \\ \end{array} .\begin{array}{*{20}c} {} \\ {} \\ \end{array} \cos \begin{array}{*{20}c} {} \\ {} \\ \end{array} (\frac{2\pi kn}{N})]\begin{array}{*{20}c} {} \\ {} \\ \end{array} } } \\ {} \\ \end{array} \begin{array}{*{20}c} {} & {} & {} \\ \end{array}$$

$$g_{s2} (k) = \begin{array}{*{20}c} {\begin{array}{*{20}c} {} \\ {} \\ \end{array} \sum\limits_{n = 0}^{N - 1} {[g_{s} (n)\begin{array}{*{20}c} {} \\ {} \\ \end{array} .\begin{array}{*{20}c} {} \\ {} \\ \end{array} \sin \begin{array}{*{20}c} {} \\ {} \\ \end{array} (\frac{2\pi kn}{N})]\begin{array}{*{20}c} {} \\ {} \\ \end{array} } } \\ {} \\ \end{array} \begin{array}{*{20}c} {} & {} & {} \\ \end{array}$$

$$g_{x1} (k) = \begin{array}{*{20}c} {\begin{array}{*{20}c} {} \\ {} \\ \end{array} \sum\limits_{n = 0}^{N - 1} {[g_{x} (n)\begin{array}{*{20}c} {} \\ {} \\ \end{array} .\begin{array}{*{20}c} {} \\ {} \\ \end{array} \cos \begin{array}{*{20}c} {} \\ {} \\ \end{array} (\frac{2\pi kn}{N})]\begin{array}{*{20}c} {} \\ {} \\ \end{array} } } \\ {} \\ \end{array} \begin{array}{*{20}c} {} & {} & {} \\ \end{array}$$

$$g_{x2} (k) = \begin{array}{*{20}c} {\begin{array}{*{20}c} {} \\ {} \\ \end{array} \sum\limits_{n = 0}^{N - 1} {[g_{x} (n)\begin{array}{*{20}c} {} \\ {} \\ \end{array} .\begin{array}{*{20}c} {} \\ {} \\ \end{array} \sin \begin{array}{*{20}c} {} \\ {} \\ \end{array} (\frac{2\pi kn}{N})]\begin{array}{*{20}c} {} \\ {} \\ \end{array} } } \\ {} \\ \end{array} \begin{array}{*{20}c} {} & {} & {} \\ \end{array}$$

*Cg*_*sx*_*(k)*: The cross-coherence estimator, on a given frequency (*k*), computed between the two sampled waves (*g*_*s*_*(n)* and* g*_*x*_*(n)*) for the two phases *‘S’* and* ‘X’,* respectively,

*g*_*s1*_*(k):* Cosine part of the DFT for the instantaneous values of *g*_*s*_*(n),*

*g*_*s2*_*(k):* Sine part of the DFT for the instantaneous values of *g*_*s*_*(n),*

*g*_*x1*_*(k):* Cosine part of the DFT for the instantaneous values of *g*_*x*_*(n),*

*g*_*x2*_*(k):* Sine part of the DFT for the instantaneous values of *g*_*x*_*(n),*

Equation 1 can be applied to obtain the following six cross-coherence estimators: *Cv*_*ab*_*(k), Cv*_*bc*_*(k), Cv*_*ca*_*(k), Ci*_*ab*_*(k), Ci*_*bc*_*(k),* and *Ci*_*ca*_*(k)*. Each cross-coherence coefficient is computed between each two corresponding data sets of the two electrical waves.

#### Auto-coherence estimator of each phase signal

The auto-coherence estimator (*Cg*_*s*_) can be found between each two successive data sets shifted from each other by one cycle of the *‘S’* phase signal (*g*_*s*_*(n-N*_*s*_*)* and* g*_*s*_*(n)*), as follows^43^:

2 $$Cg_{s} (k) = \frac{{\left[ {} \right.(\begin{array}{*{20}c} {} \\ {} \\ \end{array} \sum\limits_{n = 0}^{N - 1} {g_{s1} (k) \times g_{s3} (k)} + g_{s2} (k) \times g_{s4} (k)\begin{array}{*{20}c} {} \\ {} \\ \end{array} )^{2} + \begin{array}{*{20}c} {} \\ {} \\ \end{array} (\begin{array}{*{20}c} {} \\ {} \\ \end{array} \sum\limits_{n = 0}^{N - 1} {g_{s1} } (k) \times g_{s4} (k) - g_{s2} (k) \times g_{s3} (k)\begin{array}{*{20}c} {} \\ {} \\ \end{array} )^{2} \left. {} \right]}}{{\sum\limits_{n = 0}^{N - 1} {} \left[ {} \right.(\begin{array}{*{20}c} {} \\ {} \\ \end{array} g_{s1} (k)\begin{array}{*{20}c} {} \\ {} \\ \end{array} )^{2} + (\begin{array}{*{20}c} {} \\ {} \\ \end{array} g_{s2} (k)\begin{array}{*{20}c} {} \\ {} \\ \end{array} )^{2} \left. {} \right] \times \sum\limits_{n = 0}^{N - 1} {} \left[ {} \right.(\begin{array}{*{20}c} {} \\ {} \\ \end{array} g_{s3} (k)\begin{array}{*{20}c} {} \\ {} \\ \end{array} )^{2} + (\begin{array}{*{20}c} {} \\ {} \\ \end{array} g_{s4} (k)\begin{array}{*{20}c} {} \\ {} \\ \end{array} )^{2} \left. {} \right]}}\begin{array}{*{20}c} {} & {} & {} \\ \end{array}$$

where,

$$g_{s3} (k) = \begin{array}{*{20}c} {\begin{array}{*{20}c} {} \\ {} \\ \end{array} \sum\limits_{n = 0}^{N - 1} {[g_{s} (n - N_{s} )\begin{array}{*{20}c} {} \\ {} \\ \end{array} .\begin{array}{*{20}c} {} \\ {} \\ \end{array} \cos \begin{array}{*{20}c} {} \\ {} \\ \end{array} (\frac{2\pi kn}{N})]\begin{array}{*{20}c} {} \\ {} \\ \end{array} } } \\ {} \\ \end{array} \begin{array}{*{20}c} {} & {} & {} \\ \end{array}$$

$$g_{s4} (k) = \begin{array}{*{20}c} {\begin{array}{*{20}c} {} \\ {} \\ \end{array} \sum\limits_{n = 0}^{N - 1} {[g_{s} (n - N_{s} )\begin{array}{*{20}c} {} \\ {} \\ \end{array} .\begin{array}{*{20}c} {} \\ {} \\ \end{array} \sin \begin{array}{*{20}c} {} \\ {} \\ \end{array} (\frac{2\pi kn}{N})]\begin{array}{*{20}c} {} \\ {} \\ \end{array} } } \\ {} \\ \end{array} \begin{array}{*{20}c} {} & {} & {} \\ \end{array}$$

*Cg*_*s*_*(k):* The auto-coherence estimator, on a given frequency (*k*), quantified between each two successive data sets shifted from each other by one-cycle time of the phase signal* (g*_*s*_*(n)* and* g*_*s*_*(n—N*_*s*_*))* of the *‘S’* phase*,*

*g*_*s3*_*(k):* Cosine part of the DFT for the instantaneous values of *g*_*s*_*(n-N*_*s*_*),*

*g*_*s4*_*(k):* Sine part of the DFT for the instantaneous values of *g*_*s*_*(n-N*_*s*_*),*

Equation 2 can be used to calculate the following six auto-coherence estimators: *Cv*_*a*_*(k), Cv*_*b*_*(k)*, *Cv*_*c*_*(k)*, *Ci*_*a*_*(k), Ci*_*b*_*(k)*, and *Ci*_*c*_*(k)*.

#### Cross-coherence estimator between each phase voltage and current

The cross-coherence estimator (*Cgf*_*s*_) can be quantified between each phase voltage and current curves (*g*_*s*_*(n)* and* f*_*s*_*(n)*) of the same phase *‘S’*, as given below^43–47^:

3 $$Cgf_{s} (k) = \frac{{\left[ {} \right.(\begin{array}{*{20}c} {} \\ {} \\ \end{array} \sum\limits_{n = 0}^{N - 1} {g_{s1} (k) \times f_{s1} (k)} + g_{s2} (k) \times f_{s2} (k)\begin{array}{*{20}c} {} \\ {} \\ \end{array} )^{2} + \begin{array}{*{20}c} {} \\ {} \\ \end{array} (\begin{array}{*{20}c} {} \\ {} \\ \end{array} \sum\limits_{n = 0}^{N - 1} {g_{s1} } (k) \times f_{s2} (k) - g_{s2} (k) \times f_{s1} (k)\begin{array}{*{20}c} {} \\ {} \\ \end{array} )^{2} \left. {} \right]}}{{\sum\limits_{n = 0}^{N - 1} {} \left[ {} \right.(\begin{array}{*{20}c} {} \\ {} \\ \end{array} g_{s1} (k)\begin{array}{*{20}c} {} \\ {} \\ \end{array} )^{2} + (\begin{array}{*{20}c} {} \\ {} \\ \end{array} g_{s2} (k)\begin{array}{*{20}c} {} \\ {} \\ \end{array} )^{2} \left. {} \right] \times \sum\limits_{n = 0}^{N - 1} {} \left[ {} \right.(\begin{array}{*{20}c} {} \\ {} \\ \end{array} f_{s1} (k)\begin{array}{*{20}c} {} \\ {} \\ \end{array} )^{2} + (\begin{array}{*{20}c} {} \\ {} \\ \end{array} f_{s2} (k)\begin{array}{*{20}c} {} \\ {} \\ \end{array} )^{2} \left. {} \right]}}\begin{array}{*{20}c} {} & {} & {} \\ \end{array}$$

where,

$$f_{s1} (k) = \begin{array}{*{20}c} {\begin{array}{*{20}c} {} \\ {} \\ \end{array} \sum\limits_{n = 0}^{N - 1} {[f_{s} (n)\begin{array}{*{20}c} {} \\ {} \\ \end{array} .\begin{array}{*{20}c} {} \\ {} \\ \end{array} \cos \begin{array}{*{20}c} {} \\ {} \\ \end{array} (\frac{2\pi kn}{N})]\begin{array}{*{20}c} {} \\ {} \\ \end{array} } } \\ {} \\ \end{array} \begin{array}{*{20}c} {} & {} & {} \\ \end{array}$$

$$f_{s2} (k) = \begin{array}{*{20}c} {\begin{array}{*{20}c} {} \\ {} \\ \end{array} \sum\limits_{n = 0}^{N - 1} {[f_{s} (n)\begin{array}{*{20}c} {} \\ {} \\ \end{array} .\begin{array}{*{20}c} {} \\ {} \\ \end{array} \sin \begin{array}{*{20}c} {} \\ {} \\ \end{array} (\frac{2\pi kn}{N})]\begin{array}{*{20}c} {} \\ {} \\ \end{array} } } \\ {} \\ \end{array} \begin{array}{*{20}c} {} & {} & {} \\ \end{array}$$

*Cgf*_*s*_*(k)*: The cross-coherence estimator, on a given frequency (*k*), computed between the two corresponding data sets of the two waves* (g*_*s*_*(n)* and* f*_*s*_*(n))* for the phase ’*S*’,

*f*_*s1*_*(k):* Cosine term of the DFT for the instantaneous values of *f*_*s*_*(n),*

*f*_*s2*_*(k):* Sine term of the DFT for the instantaneous values of *f*_*s*_*(n),*

Equation 3 can be used to quantify the following three cross-coherence estimators: *Cvi*_*a*_*(k), Cvi*_*b*_*(k),* and *Cvi*_*c*_*(k)*. Each estimator is computed between each two corresponding data sets of the voltage and current waves for the same phase. The equation can identify the running power factor of the power network using the root of the cross-coherence estimator computed between the voltage and current per each phase. This principle can be a base for an automatic power factor correction (APFC). If the three cross-coherence estimators (*Cvi*_*a*_*(k), Cvi*_*b*_*(k),* and *Cvi*_*c*_*(k)*) are equal, this confirms that the system situation is normal. Otherwise, it is abnormal.

According to the conditions given in Tables 1 and 2, the proposed method can detect precisely the events of the unbalanced/abnormal voltages and currents in the three-phase power system using the fifteen coherence estimators obtained at each data set. The pre-determined data set (*N*) can control the algorithm operating time, and can be selected to be one-cycle or sub-cycle time. This means that the data set can be modified to be one, 3/4, 1/2, or 1/4 of the periodic cycle, according to the priority of protection requirements.

**Table 1:** The conditions for three-phase voltages/currents imbalance detection based on the cross-coherence and the protection action.

Sub-algorithm description	Coherence estimators domain	Generator situation (balance/imbalance condition)	The protection action (operating/blocking)
	The cross-coherence setting deviations (*Δ*_*1*_, *Δ*_*2*_ and *Δ*_*3*_) are 0.1, 0.1 and 0.1, respectively		
Sub-algorithm (1): to find the voltage imbalance conditions using the cross-coherence estimators for three-phase voltages	*0.25*+*Δ*_*1*_ ≥ *Cv*_*ab*_ ≥ 0.25-*Δ*_*1*_, *0.25*+*Δ*_*1*_ ≥ *Cv*_*bc*_ ≥ *0.25*-*Δ*_*1*_ and *0.25*+*Δ*_*1*_ ≥ *Cv*_*ca*_ ≥ *0.25*-*Δ*_*1*_	Normal and balanced voltages	Blocking action
	*0.25*+*Δ*_*1*_ < *Cv*_*ab*_ < *0.25*-*Δ*_*1*_, *0.25*+*Δ*_*1*_ < *Cv*_*bc*_ < *0.25*-*Δ*_*1*_ or *0.25*+*Δ*_*1*_ < *Cv*_*ca*_ < *0.25*-*Δ*_*1*_	Unacceptable unbalance for three phase voltages	Cooling system operation/Tripping action for SG CBs
Sub-algorithm (2): to find out the current imbalance conditions using the cross-coherence estimators for three-phase currents	*0.25*+*Δ*_*2*_ ≥ *Ci*_*ab*_ ≥ *0.25*-*Δ*_*2*_, *0.25*+*Δ*_*2*_ ≥ *Ci*_*bc*_ ≥ *0.25*-*Δ*_*2*_ and *0.25*+*Δ*_*2*_ ≥ *Ci*_*ca*_ ≥ *0.25*-*Δ*_*2*_	Normal and balanced currents	Blocking action
	*0.25*+*Δ*_*2*_ < *Ci*_*ab*_ < *0.25*-*Δ*_*2*_, *0.25*+*Δ*_*2*_ < *Ci*_*bc*_ < *0.25*-*Δ*_*2*_ or *0.25*+*Δ*_*2*_ < *Ci*_*ca*_ < *0.25*-*Δ*_*2*_	Unacceptable unbalance for three phase currents	Cooling system operation/Tripping action for SG CBs
Sub-algorithm (3): to figure out the disturbance of power factor using the cross-coherence estimators for phase voltage and current waves	*1* ≥ *Cvi*_*a*_ ≥ *1*- *Δ*_*3*_, *1* ≥ *Cvi*_*b*_ ≥ *1*- *Δ*_*3*_ and *1* ≥ *Cvi*_*c*_ ≥ *1*- *Δ*_*3*_	Normal and acceptable PF	Blocking action
	*0* < *Cvi*_*a*_ < *1*- *Δ*_*3*_, *0* < *Cvi*_*b*_ < *1*- *Δ*_*3*_ or *0* < *Cvi*_*c*_ < *1*- *Δ*_*3*_	Abnormal and unacceptable PF	Cooling system operation/Tripping action for SG CBs

**Table 2:** The conditions for phase voltage/current disturbance detection based on the auto-coherence and the protection action.

Sub-algorithm description	Coherence estimators domain	Generator situation (normal/abnormal condition)	The protection action (operating/blocking)
	The auto-coherence setting deviations (*Δ*_*4*_ and *Δ*_*5*_) are 0.1 and 0.1, respectively.		
Sub-algorithm (4): to identify the voltage disturbance using the auto-coherence estimator for each phase voltage wave	*1* ≥ *Cv*_*a*_ ≥ *1*-*Δ*_*4*_, *1* ≥ *Cv*_*b*_ ≥ *1*-*Δ*_*4*_, and *1* ≥ *Cv*_*c*_ ≥ *1*-*Δ*_*4*_	Normal and acceptable three phase voltages	Blocking action
	*0* < *Cv*_*a*_ < *1*- *Δ*_*4*_, *0* < *Cv*_*b*_ < *1*- *Δ*_*4*_ or *0* < *Cv*_*c*_ < *1*- *Δ*_*4*_	Abnormal voltage	Alarm/Tripping action for SG CBs
Sub-algorithm (5): to determine the current disturbance using the auto-coherence estimator for each phase current wave	*1* ≥ *Ci*_*a*_ ≥ *1*-*Δ*_*5*_, *1* ≥ *Ci*_*b*_ ≥ *1*-*Δ*_*5*_, and *1* ≥ *Ci*_*c*_ ≥ *1*-*Δ*_*5*_	Normal and acceptable three phase currents	Blocking action
	*0* < *Ci*_*a*_ < *1*- *Δ*_*5*_, *0* < *Ci*_*b*_ < *1*- *Δ*_*5*_ or *0* < *Ci*_*c*_ < *1*- *Δ*_*5*_	Abnormal current	Alarm/Tripping action for SG CBs

It is well-established that any disturbance/imbalance in the current signal will result in a collapse in the voltage signal of the same phase. The occurrence of this issue will have a significant impact on the auto-coherence estimators for both voltage and current signals simultaneously. Consequently, these changes will also affect the cross-coherence estimators. In other words, the cross-coherence and auto-coherence estimators change during the fault/imbalance scenarios. It is possible to design protective relays or fault detectors with the help of coherence estimators.

### Algorithm procedure

The proposed algorithm functions as follows:

1. Measure numerical values of the three-phase voltages and currents (*v*_*s*_ and *i*_*s*_) for each phase ‘*S*’ at the load terminals of the SG stator windings,
2. Select the coherence setting deviations (*Δ*_*1*_*, Δ*_*2*_*, Δ*_*3*_*, Δ*_*4*_ and* Δ*_*5*_) and the data set quantity (*N*),
3. Compute the three cross-coherence coefficients (*Cv*_*ab*_*, Cv*_*bc*_*,* and *Cv*_*ca*_) for each two-phase voltages using Eq. 1,

In order to discriminate between the balance and imbalance conditions for the three-phase voltages, the following rules should be verified:

(I) If *Cv*_*ab*_ = *Cv*_*bc*_ = *Cv*_*ca*_ = *0.25,* this situation indicates ideal balance for the three-phase voltages,
(II) If *0.25* + *Δ*_*1*_ ≥ *Cv*_*ab*_ ≥ *0.25-Δ*_*1*_*, 0.25* + *Δ*_*1*_** ≥ ***Cv*_*bc*_ ≥ *0.25-Δ*_*1*_ and *0.25* + *Δ*_*1*_ ≥ *Cv*_*ca*_ ≥ *0.25-Δ*_*1*_*,* this situation indicates acceptable unbalance for the three-phase voltages,
(III) If *0.25* + *Δ*_*1*_ <* Cv*_*ab*_ < *0.25-Δ*_*1*_, *0.25* + *Δ*_*1*_ < *Cv*_*bc*_ < *0.25-Δ*_*1*_ or* 0.25* + *Δ*_*1*_ < *Cv*_*ca*_ < *0.25-Δ*_*1*_*,* this situation indicates unacceptable unbalance for the three-phase voltages,

4. Calculate the three cross-coherence coefficients (*Ci*_*ab*_*, Ci*_*bc*_*,* and *Ci*_*ca*_) for each two-phase currents using Eq. 1,

In order to differentiate between the balance and imbalance conditions for the three-phase currents, the following rules should be satisfied:

(I) If *Ci*_*ab*_ = *Ci*_*bc*_ = *Ci*_*ca*_ = *0.25,* this situation denotes ideal balance for the three-phase currents,
(II) If *0.25* + *Δ*_*2*_ ≥ *Ci*_*ab*_ ≥ *0.25-Δ*_*2*_*, 0.25* + *Δ*_*2*_** ≥ ***Ci*_*bc*_ ≥ *0.25-Δ*_*2*_ and *0.25* + *Δ*_*2*_ ≥ *Ci*_*ca*_ ≥ *0.25-Δ*_*2*_*,* this situation denotes acceptable unbalance for the three-phase currents,
(III) If *0.25* + *Δ*_*2*_ < *Ci*_*ab*_ < *0.25*-*Δ*_*2*_, *0.25* + *Δ*_*2*_ < *Ci*_*bc*_ < *0.25*-*Δ*_*2*_ or *0.25* + *Δ*_*2*_ < *Ci*_*ca*_ < *0.25*-*Δ*_*2*_, this situation denotes unacceptable unbalance for the three-phase currents,

5. Estimate the three cross-coherence coefficients (*Cvi*_*a*_*, Cvi*_*b*_*,* and *Cvi*_*c*_) for each phase voltage and current using Eq. 3,

In order to recognize the disturbance in power factor angle quantified between the phase voltage and current, the following rules should be verified:

(I) If *Cvi*_*a*_ = *Cvi*_*b*_ = *Cvi*_*c*_ =  + *1.0,* this situation indicates ideal operating PF of the three-phase power system,
(II) If *1* ≥ *Cvi*_*a*_ ≥ *1-Δ*_*3*_*, 1* ≥ *Cvi*_*b*_ ≥ *1-Δ*_*3*_*,* and* 1* ≥ *Cvi*_*c*_ ≥ *1-Δ*_*3*_*,* this situation indicates normal and acceptable PF of the three-phase power system,
(III) If *0* < *Cvi*_*a*_ < *1*- *Δ*_*3*_, *0* < *Cvi*_*b*_ < *1*- *Δ*_*3*_ or *0* < *Cvi*_*c*_ < *1*- *Δ*_*3*_, this situation indicates abnormal and unacceptable PF of the three-phase power system,

6. Quantify the three auto-coherence coefficients (*Cv*_*a*_*, Cv*_*b*_*,* and *Cv*_*c*_) for each phase voltage using Eq. 2,

In order to detect the disturbance in phase voltage waveform, the following rules should be verified:

(I) If *Cv*_*a*_ = *Cv*_*b*_ = *Cv*_*c*_ =  + *1.0,* this situation denotes ideal and normal operation of the three-phase system voltages,
(II) If *1* ≥ *Cv*_*a*_ ≥ *1-Δ*_*4*_*, 1* ≥ *Cv*_*b*_ ≥ *1-Δ*_*4*_*,* and* 1* ≥ *Cv*_*c*_ ≥ *1-Δ*_*4*_*,* this situation denotes normal and acceptable operation of the three-phase system voltages,
(III) If *0* < *Cv*_*a*_ < *1*- *Δ*_*4*_, *0* < *Cv*_*b*_ < *1*- *Δ*_*4*_ or *0* < *Cv*_*c*_ < *1*- *Δ*_*4*_, this situation denotes abnormal and unacceptable operation of the three-phase system voltages,

7. Compute the three auto-coherence coefficients (*Ci*_*a*_*, Ci*_*b*_*,* and *Ci*_*c*_) for each phase current using Eq. 2,

In order to find out the disturbance in phase current waveform, the following rules should be satisfied:

(I) If *Ci*_*a*_ = *Ci*_*b*_ = *Ci*_*c*_ =  + *1.0,* this situation indicates ideal and normal operation of the three-phase system currents,
(II) If *1* ≥ *Ci*_*a*_ ≥ *1-Δ*_*5*_*, 1* ≥ *Ci*_*b*_ ≥ *1-Δ*_*5*_*,* and* 1* ≥ *Ci*_*c*_ ≥ *1-Δ*_*5*_*,* this situation indicates normal and acceptable operation of the three-phase system currents,
(III) If *0* < *Ci*_*a*_ < *1*- *Δ*_*5*_, *0* < *Ci*_*b*_ < *1*- *Δ*_*5*_ or *0* < *Ci*_*c*_ < *1*- *Δ*_*5*_, this situation indicates abnormal and unacceptable operation of the three-phase system currents,

8. The response of the proposed protection technique relies on the conditions listed in Tables 1 and 2.
9. In the present protection scheme, the following recommendations should be considered:

- The setting deviations (*Δ*_*1*_*, Δ*_*2*_, *Δ*_*3*_*, Δ*_*4*_ and* Δ*_*5*_) of the coherence estimators should be bounded within the margin of 0.0 and + 0.1 to avoid the miss-operation in the events of temporary faults, DC components and decent ripples. These deviations are pre-determined according to the prevailed conditions of the power network, protection requirements, and the permissible level of the system imbalance,
- The relay sensitivity, security, and speed are controlled via the span of the data set area (*N*) and the coherence deviation settings (*Δ*_*1*_*, Δ*_*2*_, *Δ*_*3*_*, Δ*_*4*_ and* Δ*_*5*_), and.
- The proposed approach has the ability to classify the unbalance situations into three levels: weak, moderate and severe unbalances in order to take the suitable relay action according to the severity degree of each category.
- The methodology can detect external or internal shunt faults located outside or inside the generator protection zone, respectively, using three single-phase current transformers built at the load terminals of the machine. Furthermore, when three single-phase current transformers are built at both neutral and load terminals of the AC machine windings, the numerical method based on the coherence criterion can diagnose the faults on the AC machine stator windings, such as turn-to-turn, winding-to-neutral and winding-to-winding faults.
- When the generator load is an AC motor, the starting current may exceed 8.0 times of its rated current. To distinguish between the fault current and the motor startup current, it is advisable to incorporate a time element in order to restrain the protection operation during the machine’s starting period. In the event of generator/motor faults, the actual operating time of the protection should be set beyond the motor starting time.

**Fig. 1:**
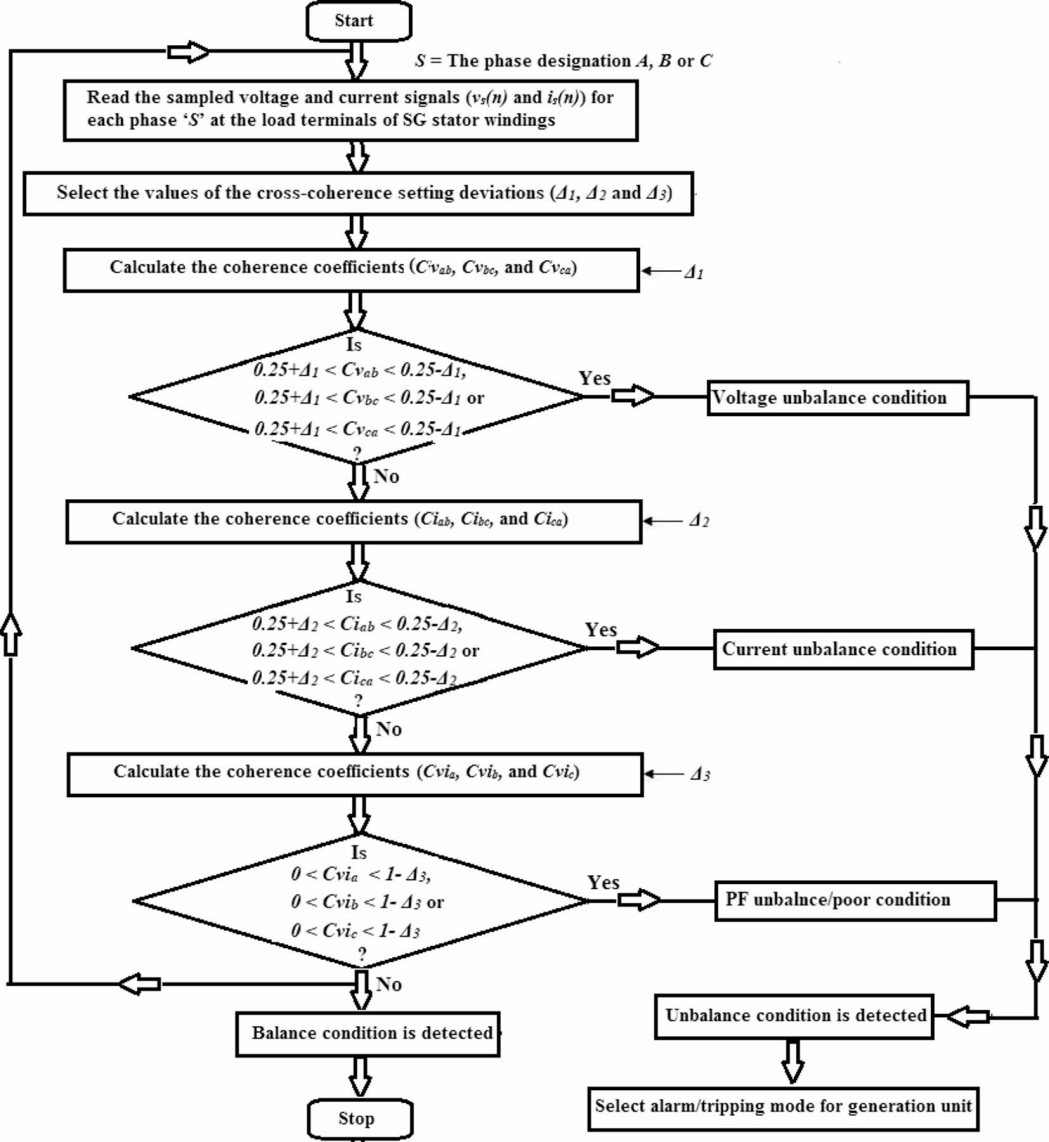

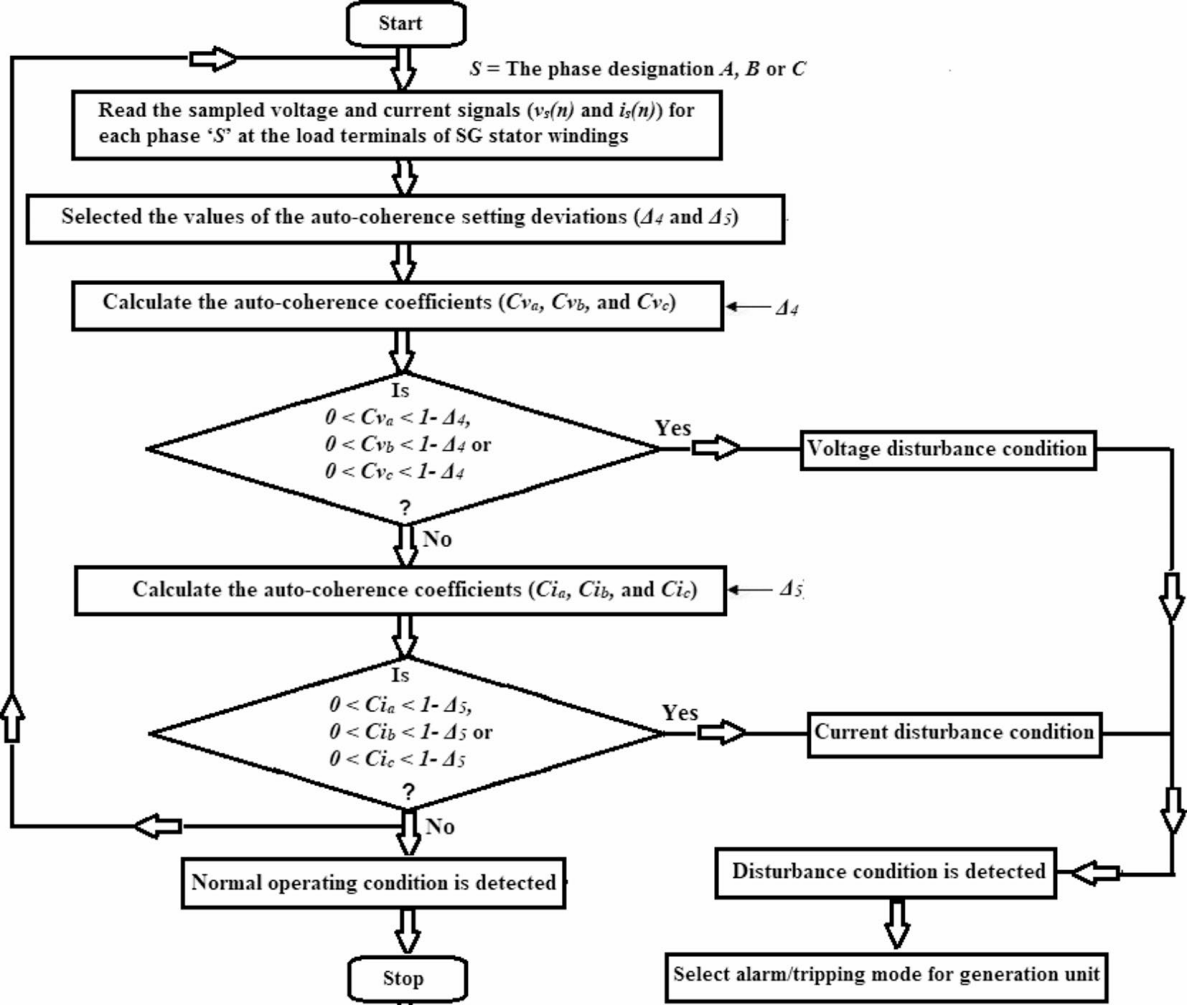
**(a)** Flow chart of the proposed algorithm for three-phase voltages and currents unbalance detection. **(b)** Flow chart of the proposed algorithm for phase voltage and current disturbance detection.

The flow chart of the proposed algorithm for three-phase voltage and current unbalance detection is shown Fig. 1a, while the flow chart of the proposed algorithm for phase voltage and current disturbance detection is depicted Fig. 1b. The two flow charts are sequentially processed. Five sub-algorithms run in series using fifteen coherence estimators (i.e., nine cross-coherence estimators and six auto-coherence estimators) quantified for six electrical variables (i.e., three-phase voltages and currents). As a result, the present technique is considered to be a highly integrated protection system.

### Imbalance and disturbance indicators

It is known that an Absolute Error (*AE*) can be calculated by subtracting an ideal value from a measured value. The Maximum Absolute Error (*MAE*) can be used to obtain two imbalance indicators (*Uv *and* Ui*) for the coherence estimators of the three-phase voltages and currents, and three disturbance indicators (*Dvi*_*,*_* Dv* and* Di*) for the coherence estimators of the single-phase voltage and current. Table 3 contains mathematical equations for evaluating the five indicators (*Uv*,* Ui, Dvi*_*,*_* Dv* and* Di*). In this proposal, the *MAE* is a dimensionless quantity since the coherence estimators are without a measurement unit.

**Table 3:** Imbalance and disturbance indicators.

Imbalance indicator for three-phase voltage coherences (*Uv*)	$$Uv = \begin{array}{*{20}c} {} \\ {} \\ \end{array} Max\left\{ {} \right.\left| {} \right.Cv_{ab} - 0.25\left. {} \right|,\begin{array}{*{20}c} {} \\ {} \\ \end{array} \left| {} \right.Cv_{bc} - 0.25\left. {} \right|\begin{array}{*{20}c} {} \\ {} \\ \end{array} or\left| {} \right.Cv_{ca}^{{}} - 0.25\left. {} \right|$$
Imbalance indicator for three-phase current coherences (*Ui*)	$$Ui = \begin{array}{*{20}c} {} \\ {} \\ \end{array} Max\left\{ {} \right.\left| {} \right.Ci_{ab} - 0.25\left. {} \right|,\begin{array}{*{20}c} {} \\ {} \\ \end{array} \left| {} \right.Ci_{bc} - 0.25\left. {} \right|\begin{array}{*{20}c} {} \\ {} \\ \end{array} or\left| {} \right.Ci_{ca}^{{}} - 0.25\left. {} \right|$$
Disturbance indicator for single-phase voltage and current coherences (*Dvi*)	$$Dvi = \begin{array}{*{20}c} {} \\ {} \\ \end{array} Max\left\{ {} \right.\left| {} \right.Cvi_{a} - 1.0\left. {} \right|,\begin{array}{*{20}c} {} \\ {} \\ \end{array} \left| {} \right.Cvi_{b} - 1.0\left. {} \right|\begin{array}{*{20}c} {} \\ {} \\ \end{array} or\left| {} \right.Cvi_{c}^{{}} - 1.0\left. {} \right|$$
Disturbance indicator for single-phase voltage coherences (*Dv*(	$$Dv = \begin{array}{*{20}c} {} \\ {} \\ \end{array} Max\left\{ {} \right.\left| {} \right.Cv_{a} - 1.0\left. {} \right|,\begin{array}{*{20}c} {} \\ {} \\ \end{array} \left| {} \right.Cv_{b} - 1.0\left. {} \right|\begin{array}{*{20}c} {} \\ {} \\ \end{array} or\left| {} \right.Cv_{c}^{{}} - 1.0\left. {} \right|$$
Disturbance indicator for single-phase current coherences (*Di*)	$$Di = \begin{array}{*{20}c} {} \\ {} \\ \end{array} Max\left\{ {} \right.\left| {} \right.Ci_{a} - 1.0\left. {} \right|,\begin{array}{*{20}c} {} \\ {} \\ \end{array} \left| {} \right.Ci_{b} - 1.0\left. {} \right|\begin{array}{*{20}c} {} \\ {} \\ \end{array} or\left| {} \right.Ci_{c}^{{}} - 1.0\left. {} \right|$$

### Protection tripping-characteristics

**Fig. 2:**
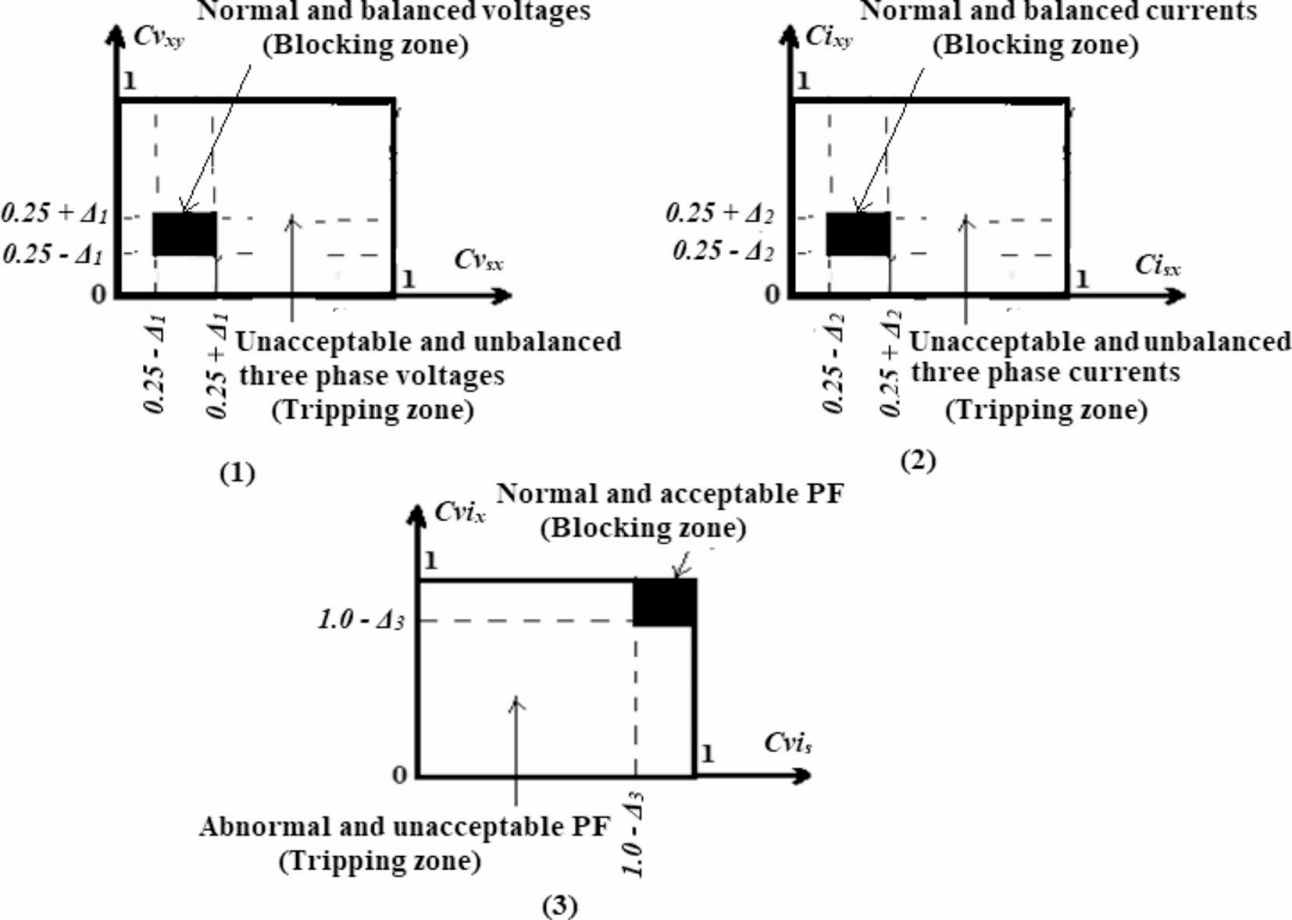

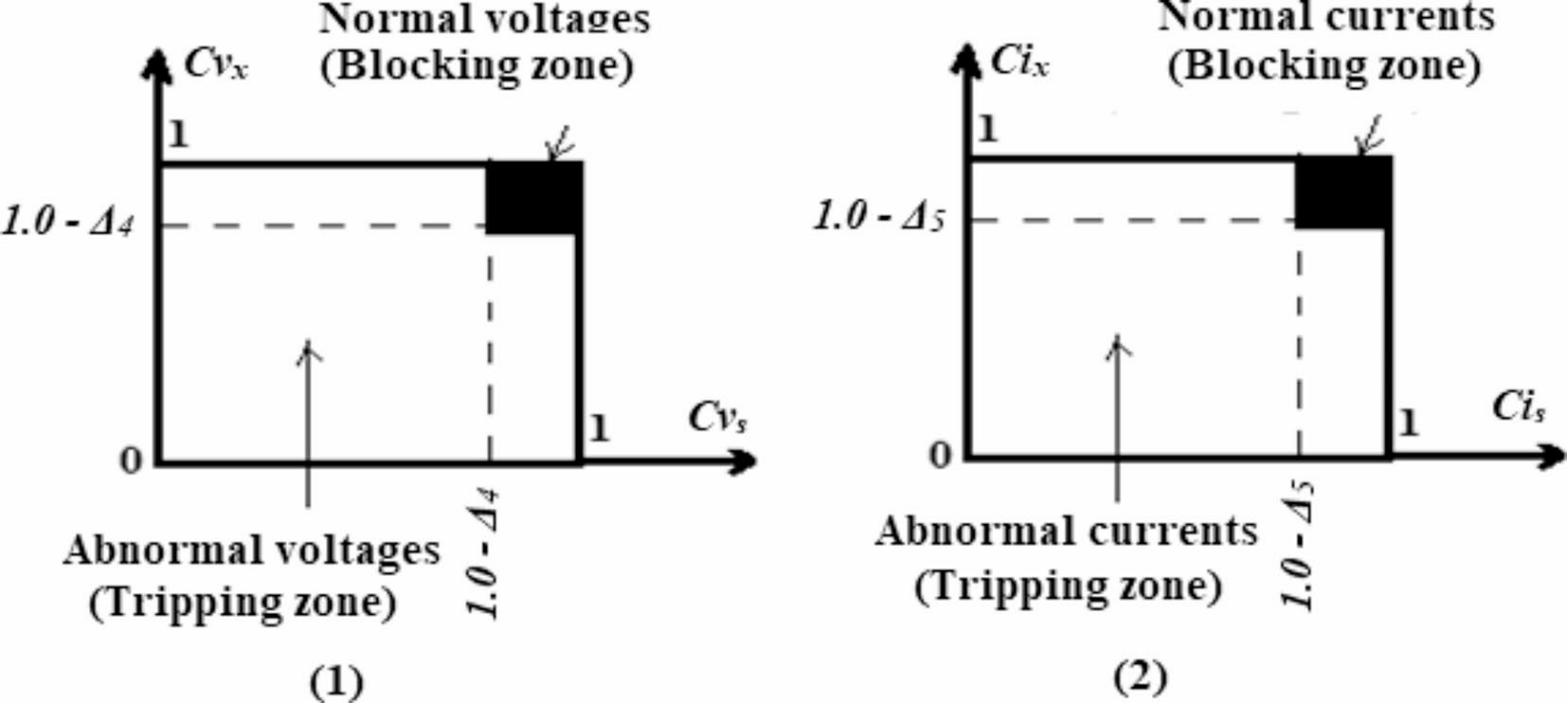
Tripping-characteristics based on **(a)** Cross-coherence coefficients and **(b)** Auto-coherence coefficients.

In this article, the authors suggest a group of tripping-characteristics for finding the imbalance and fault situations in three-phase voltage and current variables. The unbalanced voltage, current, and power factor detectors rely on the tripping-characteristics that use the nine cross-coherence coefficients, as shown in Fig. 2a. The fault detector depends on the tripping-characteristics that use the six auto-coherence coefficients that are depicted in Fig. 2b. The relay tripping-characteristics have a closed curve design, as illustrated in Fig. 2a-b, because they depend on the bound values of the coherence, which range from zero to one. During normal or abnormal operating conditions of the power grid, the operating points of the relay characteristics do not surpass this area. The outside influences/parameters and the properties of the system are involved in these points. Each tripping curve includes two regions:

(I) There is a blocking region to prevent the operation of the protection algorithm in the cases of balance and normal operation for the three-phase voltages and currents with acceptable power factor, and

(II) Additionally, there is a tripping region that allows the protection algorithm to react in the event of the unbalance, fault occurrence or both with an unacceptable power factor. When the operating points are concentrated in this region, a trip signal is issued to the generator breakers.

## Power system model under test

**Fig.3:**
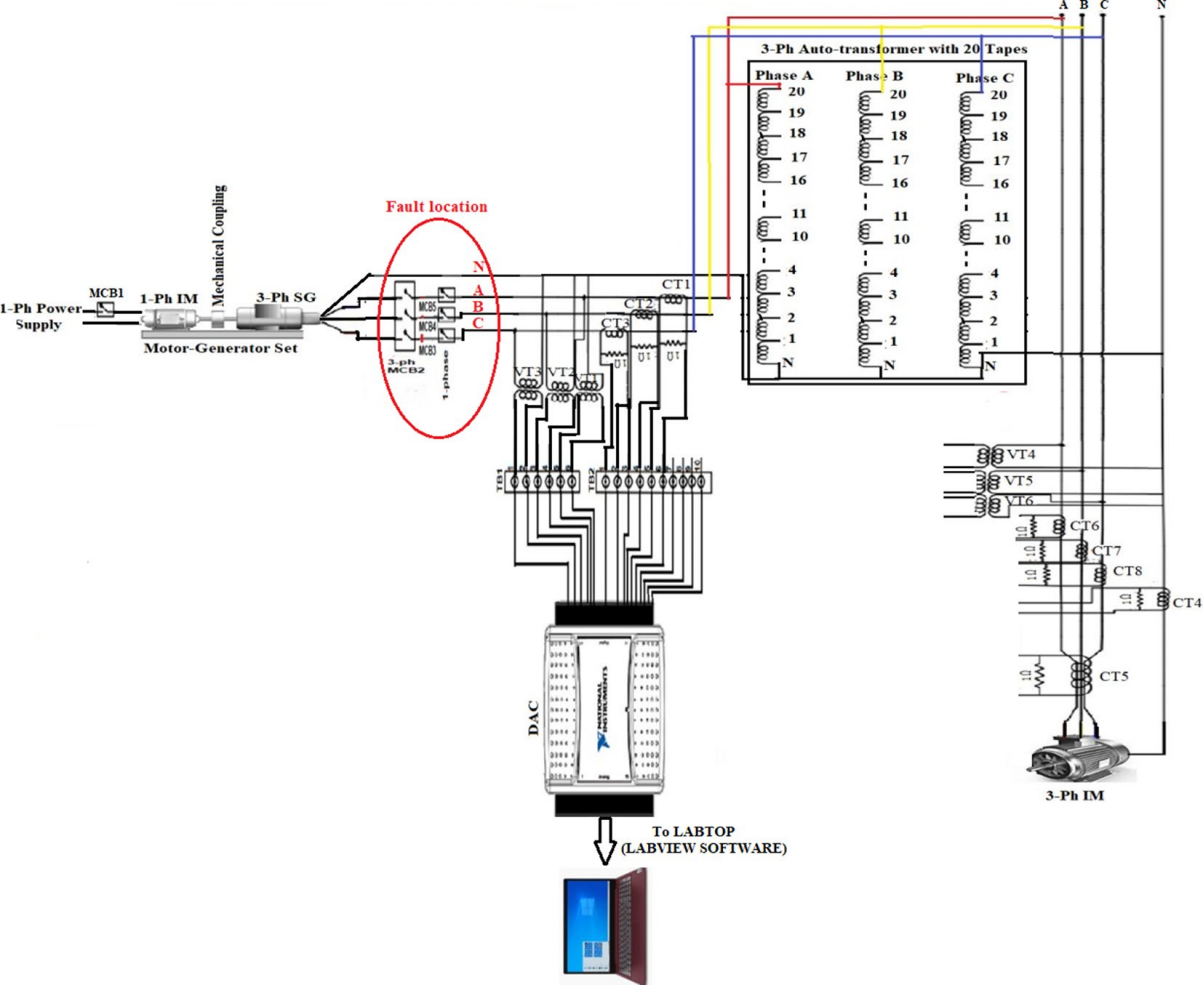

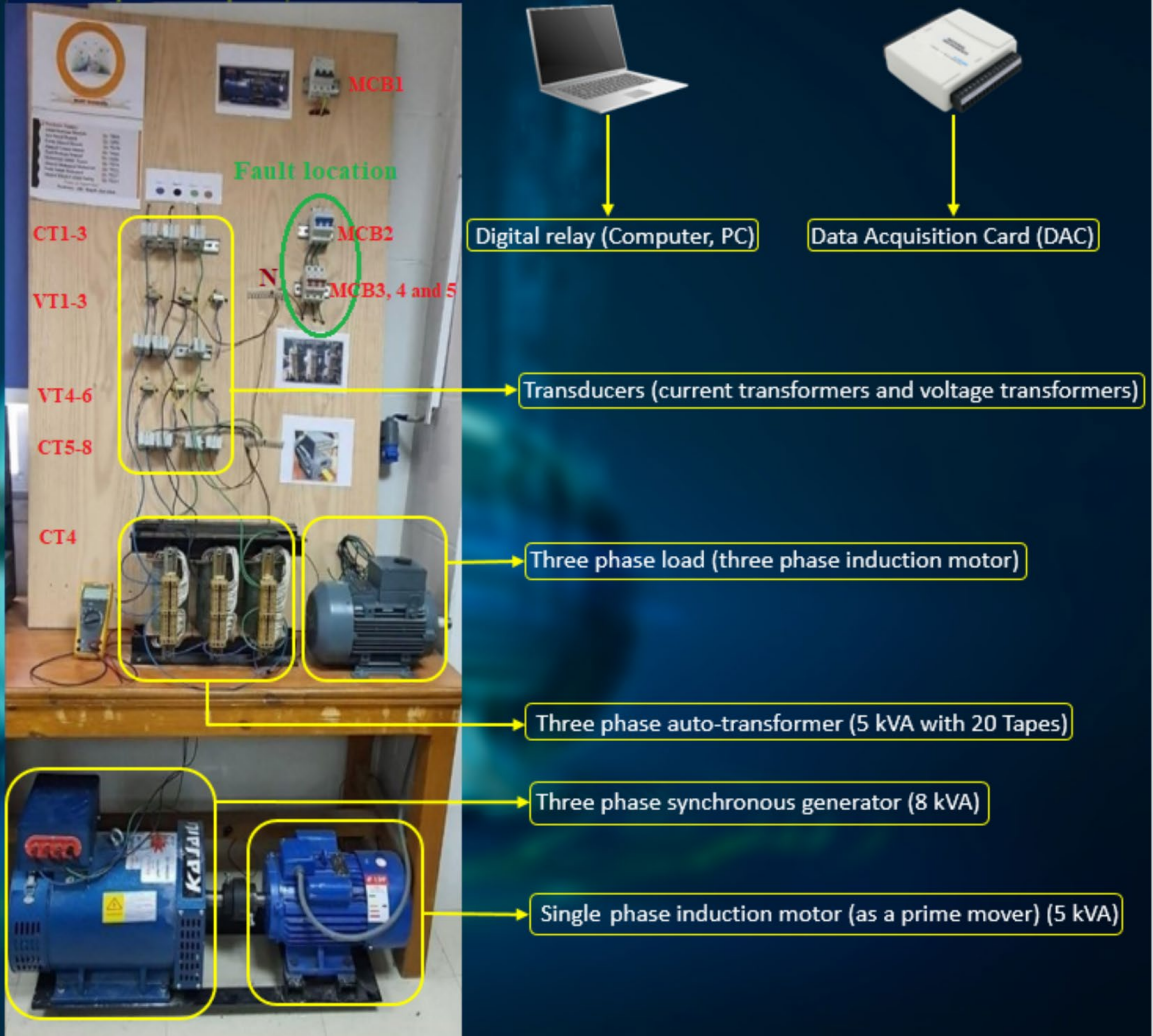
(**a**) Connection-wiring diagram of the power model. (**b**) Main components of the system model.

In this paper, the proposed algorithm is validated using an experimental model. Figure 3a shows the connection-wiring diagram for the power system model under test. The real photo of the experimental model is shown in Fig. 3b. A motor-generator (M-G) system under test is a combination of a single-phase induction motor and a three-phase synchronous generator (SG(, which are mechanically coupled through a common shaft. The generator is equipped with a three-phase induction motor that represents the electrical load. As shown in Fig. 3a-b, three-phase voltage transformers (VT_1_, VT_2_ and VT_3_) and three-phase current transformers (CT_1_, CT_2_ and CT_3_) are built at the terminals of the SG stator windings for measuring three-phase voltages (*v*_*a*_*, v*_*b*_ and *v*_*c*_) and three-phase currents (*i*_*a*_*, i*_*b*_ and *i*_*c*_), respectively. Appendix 1 provides the specifications for each component of the power system model.

## Results and discussions

In this article, the hardware of the intelligent digital relay is emulated utilizing a personal computer with the Data Acquisition Card (DAC). As seen in Fig. 3a-b, DAC is National Instruments USB-6008/6009 that is used to convert the analog signals into discrete values. The protection algorithm is employed under the effect of various imbalance and disturbance conditions for the six electrical signals taken at the SG terminals using both DAC and LABVIEW software. In this work, the sinusoidal signals are analyzed by converting them into a predefined number of samples. The cycle time of the 50 Hz system is divided into *N*_*s*_ = 50 samples per cycle. This means that the sampling rate (*F*_*s*_) is 2.5 kHz. The data set has *N* = *N*_*s*_ = 50 samples per cycle. The full display time is 0.2 Sec. (i.e. it is equivalent to 10 cycles) in this study. The proposed algorithm calculates the fifteen coherence coefficients for each data set, and then monitors the variation in these coefficients using the predefined rules (as shown in Tables 1 and 2) to find out the imbalance and disturbance events of voltage and current waveforms. To identify whether the three-phase voltages and currents are balanced or unbalanced and to confirm whether the system status is healthy or faulty, a delay time of only one cycle is needed. Numerous experiments are done on the practical model to verify the effectiveness of the algorithm under the effect of the different fault types (i.e., series and shunt faults).

In this work, MCB3, MCB4 and MCB5 have been used to conduct series faults to open single-phase, double-phases, or three-phases of the power supply during the system operation. Whereas, the shunt faults have been accomplished using short-circuits located at the three single-phase MCBs (i.e., MCB3, MCB4 and MCB5) with/without a neutral point. Shunt faults such as single phase-to-neutral, double phases, or three phases can be executed during the system operation. The findings obtained under normal operating conditions are illustrated in Table 4.

**Table 4:** The obtained findings under normal operating conditions.

Parameter	*V*_*a*_* (Volt)*	*V*_*b*_* (Volt)*	*V*_*c*_* (Volt)*	*I*_*a*_* (Amp)*	*I*_*b*_*(Amp)*	*I*_*c*_*(Amp)*
RMS phase primary value	221	219.5	220	5.8	4.9	5.4
RMS phase secondary value	5.02	4.99	5.000	0.097	0.081	0.090
Auto-coherence coefficients	***Cv***_***a***_	***Cv***_***b***_	***Cv***_***c***_	***Ci***_***a***_	***Ci***_***b***_	***Ci***_***c***_
	+ 1.0	+ 1.0	+ 1.0	+ 1.0	+ 1.0	+ 1.0
Cross-coherence coefficients	***Cv***_***ab***_	***Cv***_***bc***_	***Cv***_***ca***_	***Ci***_***ab***_	***Ci***_***bc***_	***Ci***_***ca***_
	+ 0.25	+ 0.25	+ 0.26	+ 0.24	+ 0.22	+ 0.35
Cross-coherence coefficients	***Cvi***_***a***_	***Cvi***_***b***_	***Cvi***_***c***_			
	0.14	0.14	0.15			

Note: The low PF due to the 3-phase IM under no load condition

### Test 1: Shunt fault (C-N)

**Fig. 4:**
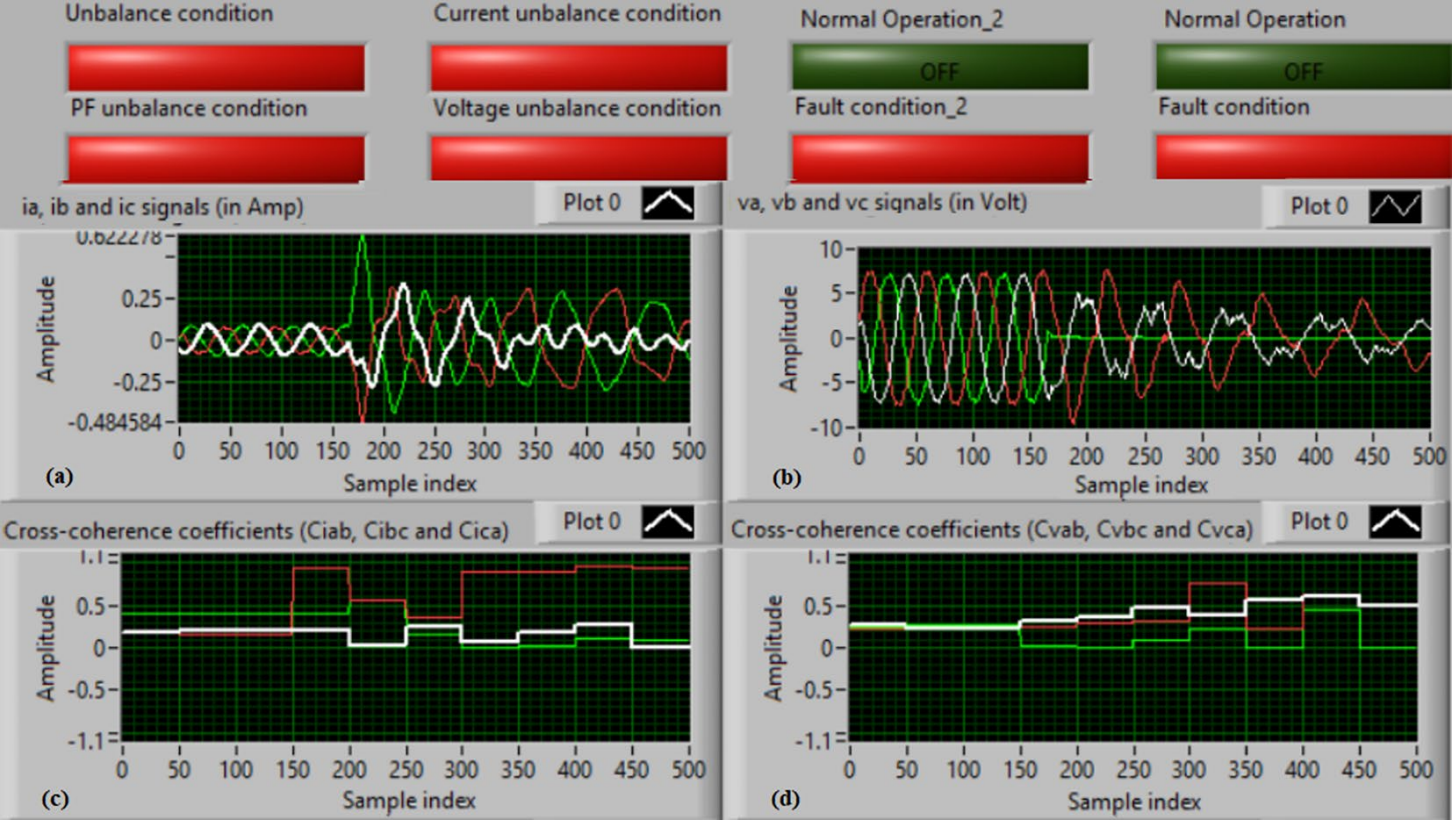
Results for experimental test 1. (**a**) Three-phase current signals* (i*_*a*_*, i*_*b*_ and *i*_*c*_*),* (**b**) Three-phase voltage signals* (v*_*a*_*, v*_*b*_ and *v*_*c*_*),* (**c**) The cross-coherence coefficients (*Ci*_*ab*_*, Ci*_*bc*_ and *Ci*_*ca*_), and (**d**) The cross-coherence coefficients (*Cv*_*ab*_*, Cv*_*bc*_*,* and *Cv*_*ca*_).

**Fig. 5:**
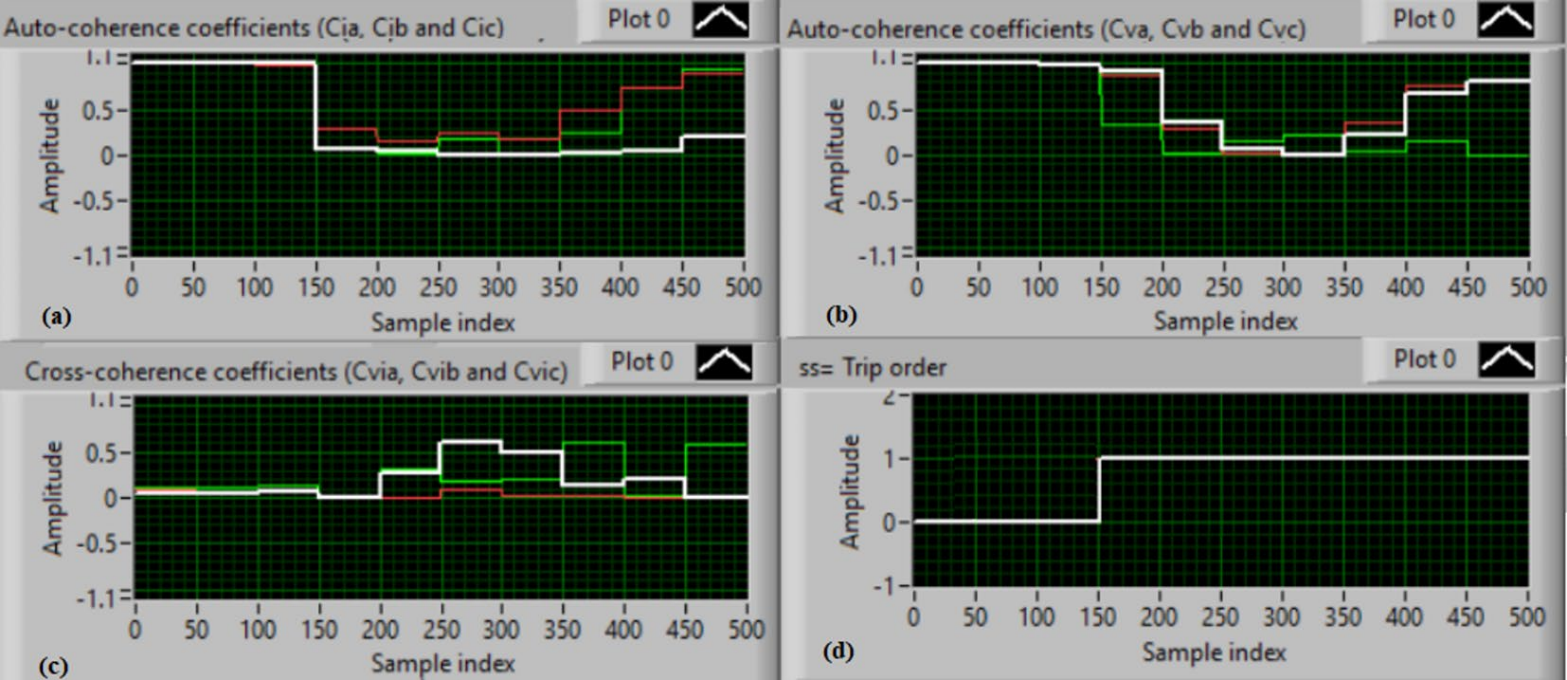
Results for experimental test 1 (Continued). (**a**) The auto-coherence coefficients (*Ci*_*a*_*, Ci*_*b*_ and *Ci*_*c*_), (**b**) The auto-coherence coefficients (*Cv*_*a*_*, Cv*_*b*_*,* and *Cv*_*c*_), (**c**) The cross-coherence coefficients (*Cvi*_*a*_*, Cvi*_*b*_*,* and *Cvi*_*c*_), and (**d**) The relay response.

For this test, the three-phase currents and voltage waveforms are shown in Fig. 4a and b, respectively. Figure 4c presents the cross-coherence coefficients (*Ci*_*ab*_*, Ci*_*bc*_*,* and *Ci*_*ca*_) of the three-phase current signals, and Fig. 4d introduces the cross-coherence coefficients (*Cv*_*ab*_*, Cv*_*bc*_*,* and *Cv*_*ca*_) of the three-phase voltage signals. Figure 5a plots the auto-coherence coefficients (*Ci*_*a*_*, Ci*_*b*_*,* and *Ci*_*c*_) of each phase current signal, and Fig. 5b shows the auto-coherence coefficients (*Cv*_*a*_*, Cv*_*b*_*,* and *Cv*_*c*_) of each phase voltage signal. Figure 5c depicts the cross-coherence coefficients (*Cvi*_*a*_*, Cvi*_*b*_*,* and *Cvi*_*c*_) computed between the phase voltage and current signals. The fifteen coherence coefficients calculated for the three-phase voltages and current are determined at each data window (i.e. one cycle). According to the rules listed in Tables 1 and 2, the coherence technique is able to detect the voltages and currents unbalance and disturbance in the power grid. It can be observed from Figs. 4c, d, 5a, b and c that when the shunt fault *(C-N)* occur, the three coherence coefficients in each figure are unequal and unstable during the fault time. Whereas, their values are settled at a fixed value before the fault occurrence. Hence, the protective relay sends a tripping order to isolate the protected machines, as shown in Fig. 5d. In this test, the red flags confirm the occurrence of current unbalance, voltage unbalance, PF disturbance, current disturbance, and voltage disturbance.

### Test 2: Shunt fault (A-B)

**Fig. 6:**
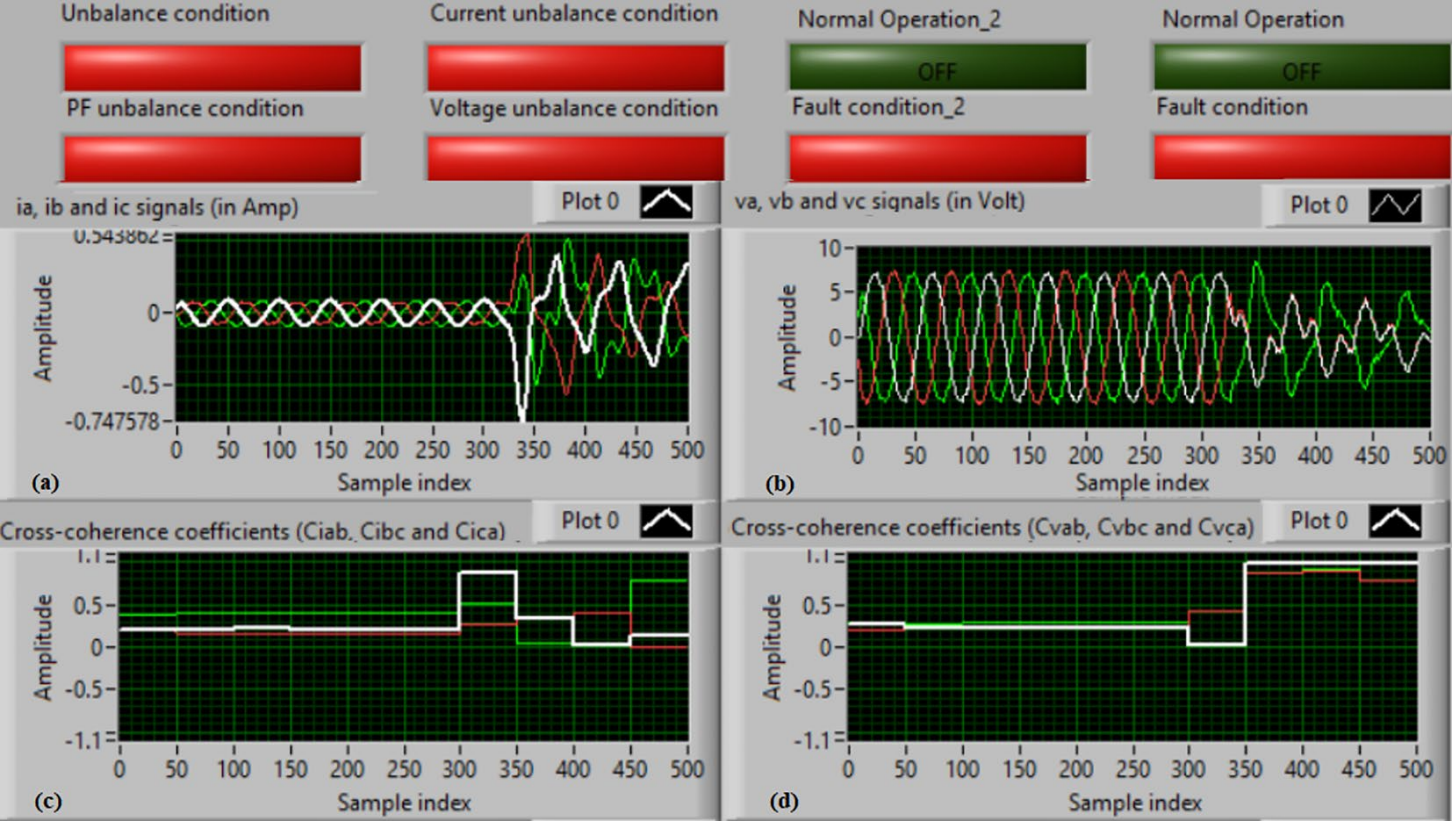
Results for experimental test 2. (**a**) Three-phase current signals* (i*_*a*_*, i*_*b*_ and *i*_*c*_*),* (**b**) Three-phase voltage signals* (v*_*a*_*, v*_*b*_ and *v*_*c*_*),* (**c**) The cross-coherence coefficients (*Ci*_*ab*_*, Ci*_*bc*_ and *Ci*_*ca*_), and (**d**) The cross-coherence coefficients (*Cv*_*ab*_*, Cv*_*bc*_*,* and *Cv*_*ca*_).

**Fig. 7:**
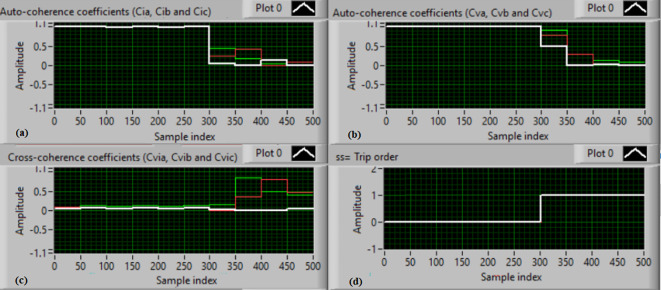
Results for experimental test 2 (Continued). (**a**) The auto-coherence coefficients (*Ci*_*a*_*, Ci*_*b*_ and *Ci*_*c*_), (**b**) The auto-coherence coefficients (*Cv*_*a*_*, Cv*_*b*_*,* and *Cv*_*c*_), (**c**) The cross-coherence coefficients (*Cvi*_*a*_*, Cvi*_*b*_*,* and *Cvi*_*c*_), and (**d**) The relay response.

For this experimental investigation, the three-phase currents and voltage signals are depicted in Fig. 6a and b, respectively. Figure 6c offers the cross-coherence coefficients (*Ci*_*ab*_*, Ci*_*bc*_*,* and *Ci*_*ca*_) of the three-phase current signals, and Fig. 6d shows the cross-coherence coefficients (*Cv*_*ab*_*, Cv*_*bc*_*,* and *Cv*_*ca*_) of the three-phase voltage signals. Figure 7a presents the auto-coherence coefficients (*Ci*_*a*_*, Ci*_*b*_*,* and *Ci*_*c*_) of each phase current signal, and Fig. 7b displays the auto-coherence coefficients (*Cv*_*a*_*, Cv*_*b*_*,* and *Cv*_*c*_) of each phase voltage signal. Figure 7c introduces the cross-coherence coefficients (*Cvi*_*a*_*, Cvi*_*b*_*,* and *Cvi*_*c*_) calculated between the phase voltage and current signals. The fifteen coherence coefficients computed for the three voltages and current are obtained at each data window. According to the rules noted in Tables 1 and 2, the coherence method has the ability to recognize the voltage and current unbalance and trouble in the power network. It can be noted from Figs. 6c, d, 7a, b and c that when the shunt fault *(A-B)* occur, the three coherence coefficients in each figure are varied and unsymmetrical during the fault interval. Whereas, their values are stationary and stable before the fault inception time. Thus, the proposed protection issues a tripping signal to isolate the protected SG, as shown in Fig. 7d. In this test, the red flags assure the events of the current unbalance, voltage unbalance, PF disturbance, current disturbance, and voltage disturbance.

### Test 3: Shunt fault (B-C)

**Fig. 8:**
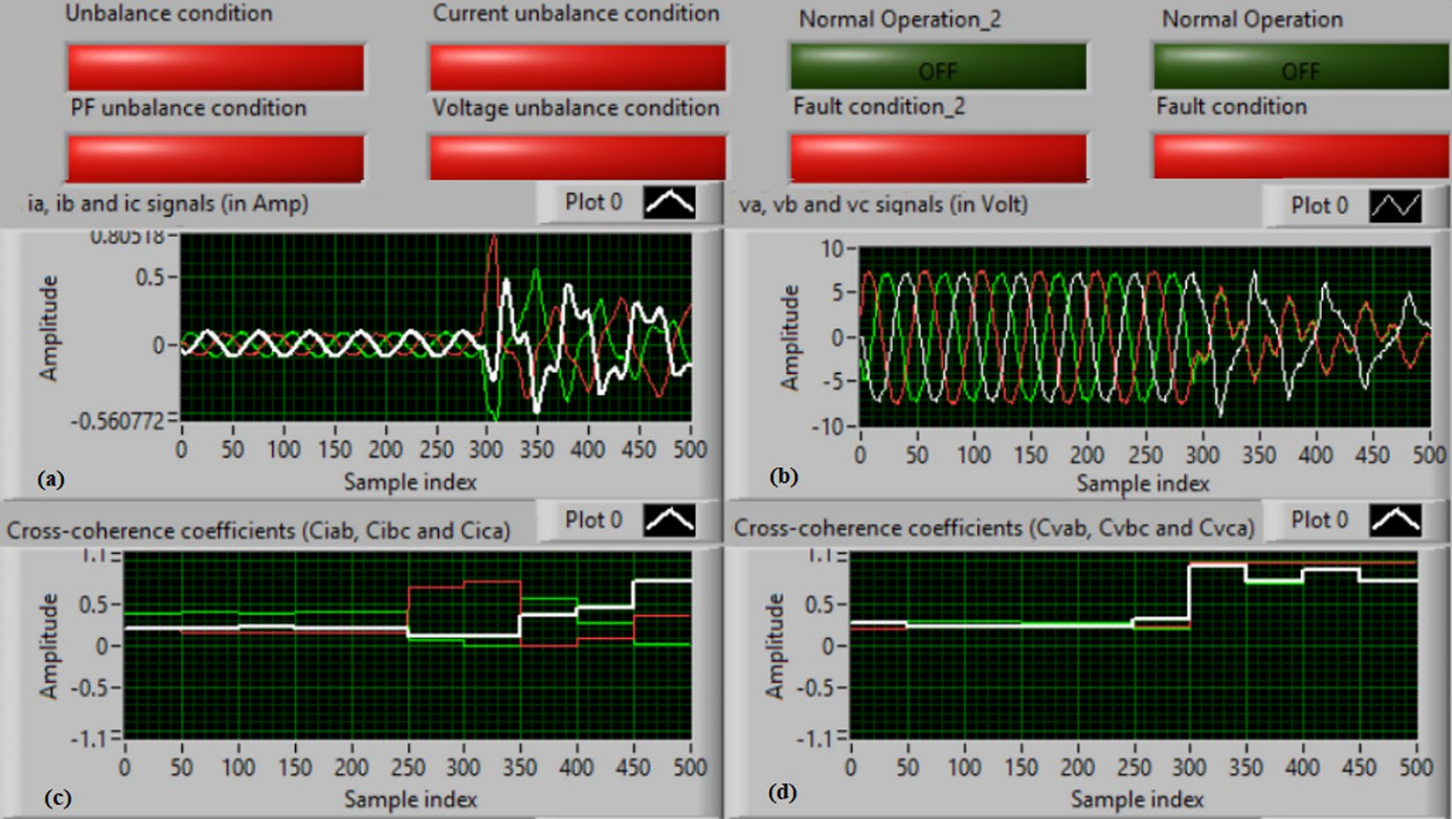
Results for experimental test 3. (**a**) Three-phase current signals* (i*_*a*_*, i*_*b*_ and *i*_*c*_*),* (**b**) Three-phase voltage signals* (v*_*a*_*, v*_*b*_ and *v*_*c*_*),* (**c**) The cross-coherence coefficients (*Ci*_*ab*_*, Ci*_*bc*_ and *Ci*_*ca*_), and (**d**) The cross-coherence coefficients (*Cv*_*ab*_*, Cv*_*bc*_*,* and *Cv*_*ca*_).

**Fig. 9:**
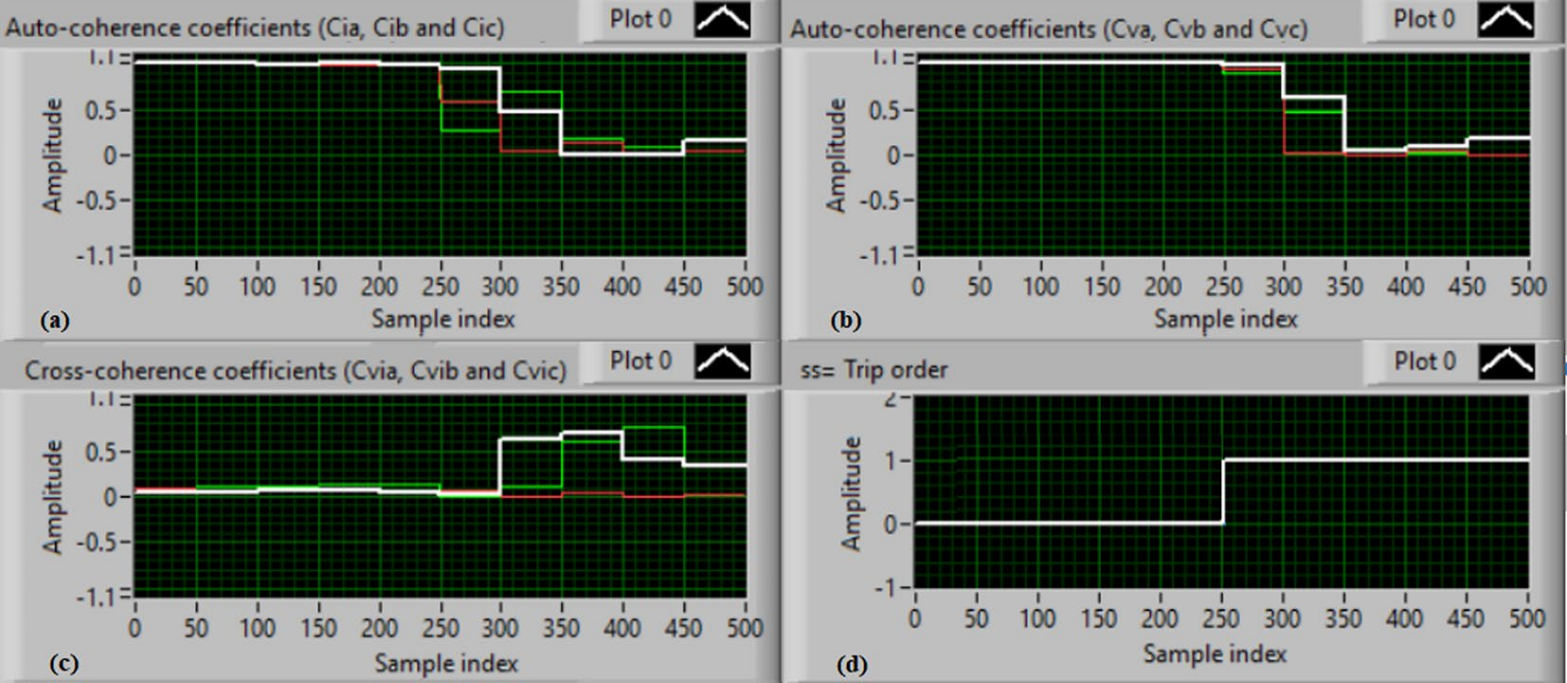
Results for experimental test 3 (Continued). (**a**) The auto-coherence coefficients (*Ci*_*a*_*, Ci*_*b*_ and *Ci*_*c*_), (**b**) The auto-coherence coefficients (*Cv*_*a*_*, Cv*_*b*_*,* and *Cv*_*c*_), (**c**) The cross-coherence coefficients (*Cvi*_*a*_*, Cvi*_*b*_*,* and *Cvi*_*c*_), and (**d**) The relay response.

For this test, the three-phase currents and voltage waveforms are shown in Fig. 8a and b, respectively. Figure 8c presents the cross-coherence coefficients (*Ci*_*ab*_*, Ci*_*bc*_*,* and *Ci*_*ca*_) of the three-phase current signals, and Fig. 8d introduces the cross-coherence coefficients (*Cv*_*ab*_*, Cv*_*bc*_*,* and *Cv*_*ca*_) of the three-phase voltage signals. Figure 9a plots the auto-coherence coefficients (*Ci*_*a*_*, Ci*_*b*_*,* and *Ci*_*c*_) of each phase current signal, and Fig. 9b shows the auto-coherence coefficients (*Cv*_*a*_*, Cv*_*b*_*,* and *Cv*_*c*_) of each phase voltage signal. Figure 9c depicts the cross-coherence coefficients (*Cvi*_*a*_*, Cvi*_*b*_*,* and *Cvi*_*c*_) calculated between the phase voltage and current signals. The fifteen coherence coefficients calculated for the three voltages and current are determined at each data window. According to the conditions listed in Tables 1 and 2, the coherence technique is able to detect the voltage and current unbalance and fault in the power grid. It is also obvious from Figs. 8c, d, 9a, b and c that when the shunt fault *(B-C)* occur, the three coherence coefficients in each figure are unequal and trouble during the fault time, while their values are settled at a fixed value before the fault occurrence. Hence, the protective relay sends a tripping order to isolate the protected generator, as shown in Fig. 9d. In this test, the red flags confirm the situations of the current unbalance, voltage unbalance, PF disturbance, current disturbance, and voltage disturbance.

### Test 4: Shunt fault (C-A)

**Fig. 10:**
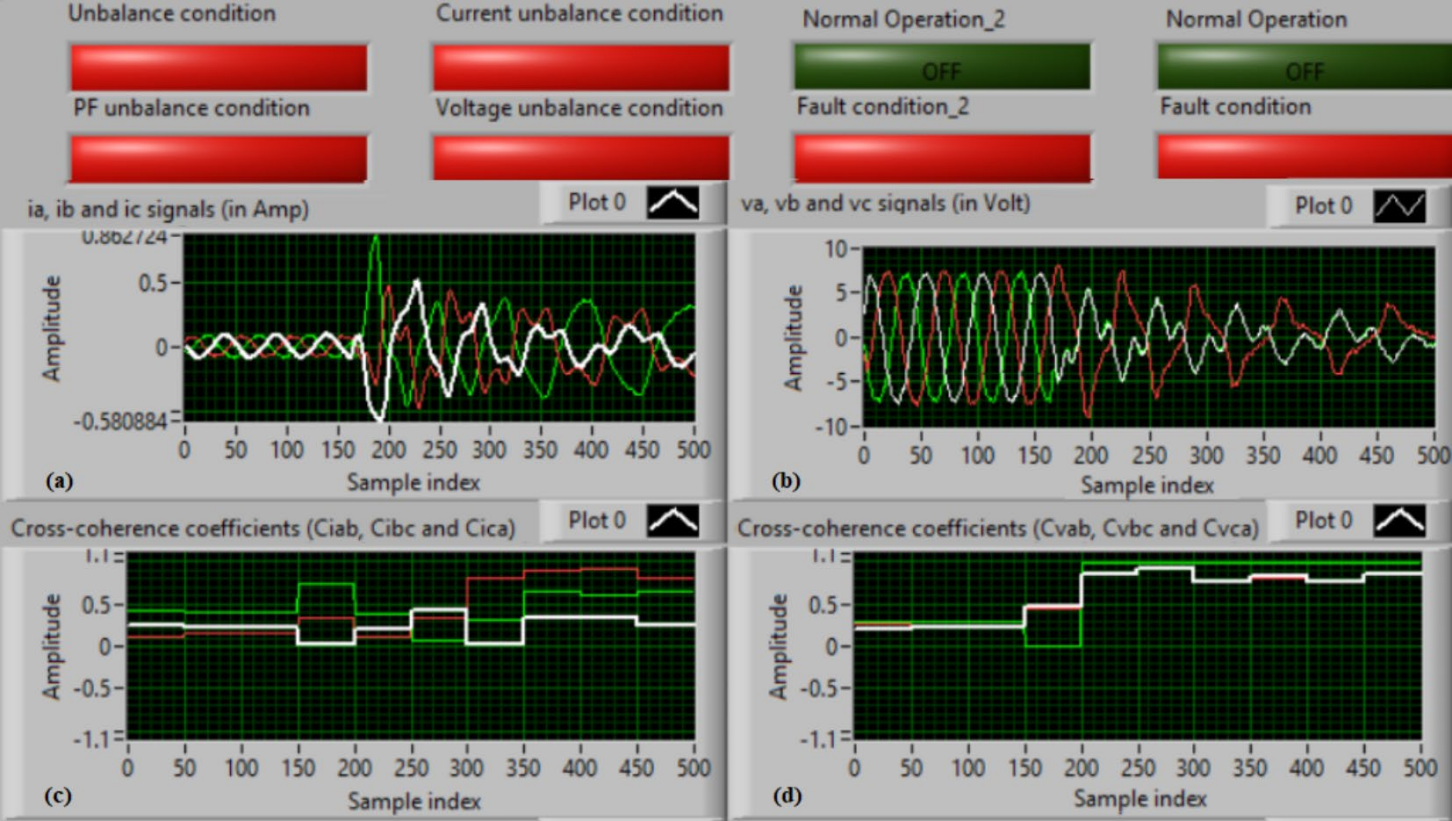
Results for experimental test 4. (**a**) Three-phase current signals* (i*_*a*_*, i*_*b*_ and *i*_*c*_*),* (**b**) Three-phase voltage signals* (v*_*a*_*, v*_*b*_ and *v*_*c*_*),* (**c**) The cross-coherence coefficients (*Ci*_*ab*_*, Ci*_*bc*_ and *Ci*_*ca*_), and (**d**) The cross-coherence coefficients (*Cv*_*ab*_*, Cv*_*bc*_*,* and *Cv*_*ca*_).

**Fig. 11:**
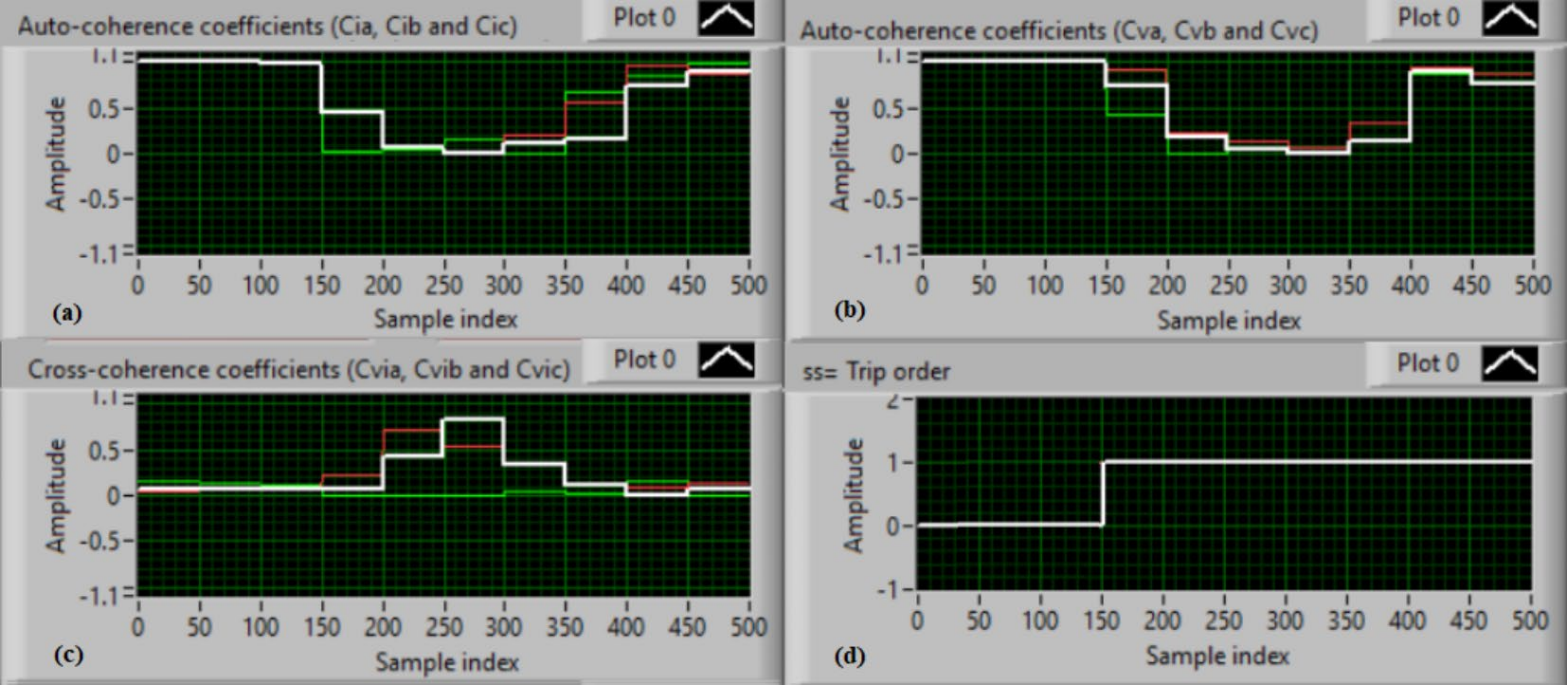
Results for experimental test 4 (Continued). (**a**) The auto-coherence coefficients (*Ci*_*a*_*, Ci*_*b*_ and *Ci*_*c*_), (**b**) The auto-coherence coefficients (*Cv*_*a*_*, Cv*_*b*_*,* and *Cv*_*c*_), (**c**) The cross-coherence coefficients (*Cvi*_*a*_*, Cvi*_*b*_*,* and *Cvi*_*c*_), and (**d**) The relay response.

For this experimental test, the three-phase currents and voltage signals are depicted in Fig. 10a and b, respectively. Figure 10c offers the cross-coherence coefficients (*Ci*_*ab*_*, Ci*_*bc*_*,* and *Ci*_*ca*_) of the three-phase current signals, and Fig. 10d shows the cross-coherence coefficients (*Cv*_*ab*_*, Cv*_*bc*_*,* and *Cv*_*ca*_) of the three-phase voltage signals. Figure 11a presents the auto-coherence coefficients (*Ci*_*a*_*, Ci*_*b*_*,* and *Ci*_*c*_) of each phase current signal, and Fig. 11b displays the auto-coherence coefficients (*Cv*_*a*_*, Cv*_*b*_*,* and *Cv*_*c*_) of each phase voltage signal. Figure 11c introduces the cross-coherence coefficients (*Cvi*_*a*_*, Cvi*_*b*_*,* and *Cvi*_*c*_) computed between the phase voltage and current signals. The fifteen coherence coefficients computed for the three voltages and current are obtained at each data window. According to the rules noted in Tables 1 and 2, the coherence method has the ability to recognize the voltage and current unbalance and fault in the power network. It can be noticed from Figs. 10c, d, 11a, b and c that when the shunt fault *(C-A)* occur, the three coherence coefficients in each figure are trouble and unsymmetrical during the fault interval. Whereas, their values are stationary and stable before the fault inception time. Thus, the proposed protection issues a tripping signal to isolate the protected SG, as given in Fig. 11d. In this test, the red flags affirm the states of the current unbalance, voltage unbalance, PF disturbance, current disturbance, and voltage disturbance. According to the practical results shown in Figs. 4, 5, 6, 7, 8, 9, 10, 11, it is reasonable to conclude that the proposed protection technique based on the fifteen coherence coefficients can detect the voltages and currents unbalance and disturbance (as a result of the shunt faults) in the three-phase power system quickly and accurately.

### Test 5: Series fault (A open)

**Fig. 12:**
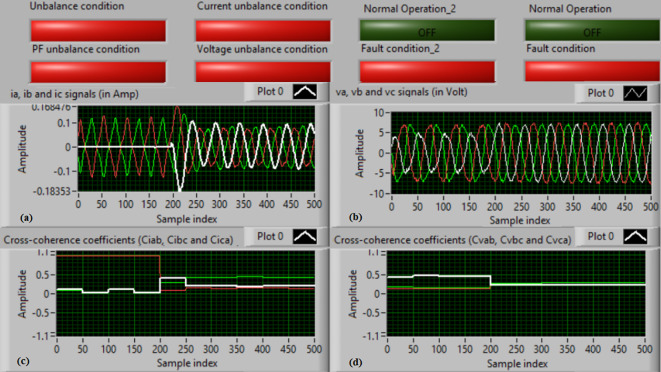
Results for experimental test 5. (**a**) Three-phase current signals* (i*_*a*_*, i*_*b*_ and *i*_*c*_*),* (**b**) Three-phase voltage signals* (v*_*a*_*, v*_*b*_ and *v*_*c*_*),* (**c**) The cross-coherence coefficients (*Ci*_*ab*_*, Ci*_*bc*_ and *Ci*_*ca*_), and (**d**) The cross-coherence coefficients (*Cv*_*ab*_*, Cv*_*bc*_*,* and *Cv*_*ca*_).

**Fig. 13:**
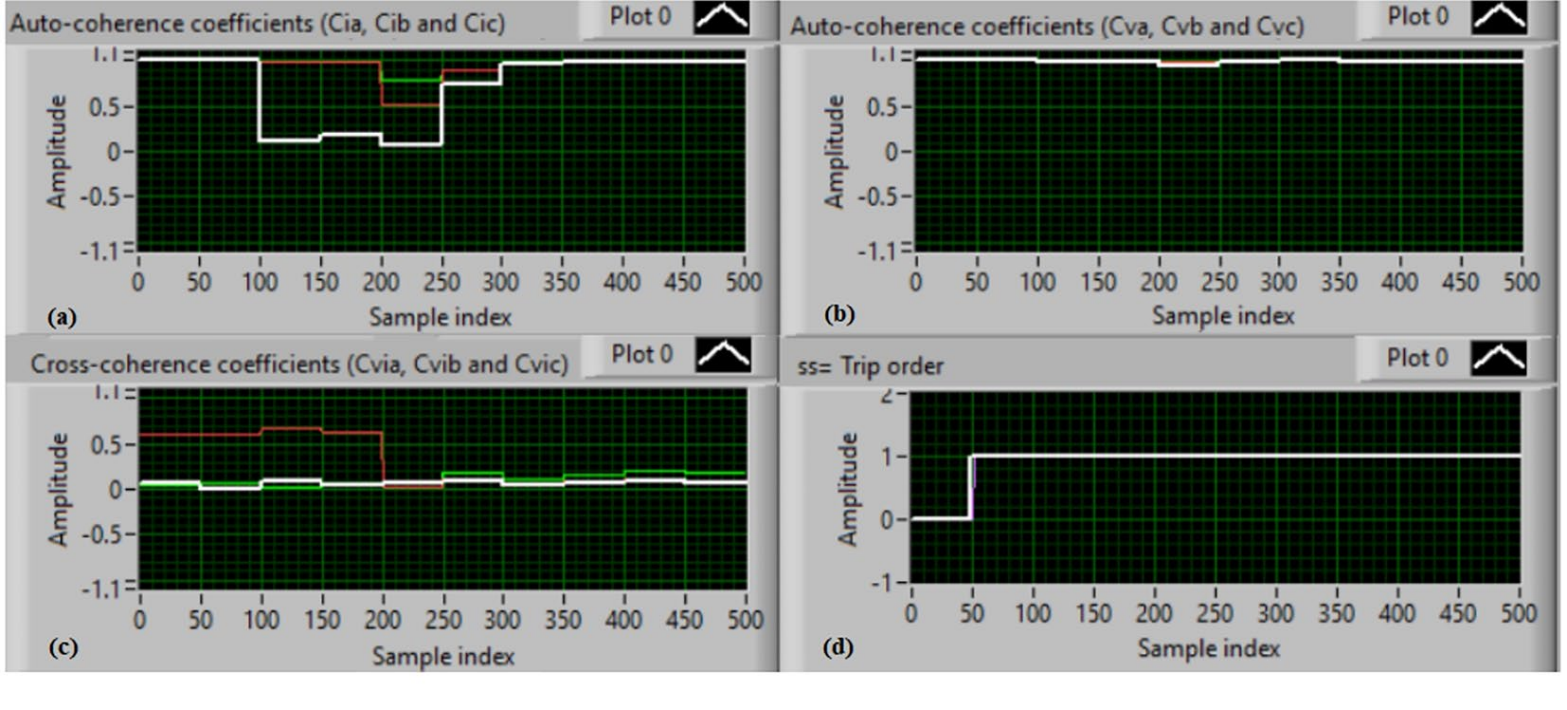
Results for experimental test 5 (Continued). (**a**) The auto-coherence coefficients (*Ci*_*a*_*, Ci*_*b*_ and *Ci*_*c*_), (**b**) The auto-coherence coefficients (*Cv*_*a*_*, Cv*_*b*_*,* and *Cv*_*c*_), (**c**) The cross-coherence coefficients (*Cvi*_*a*_*, Cvi*_*b*_*,* and *Cvi*_*c*_), and (**d**) The relay response.

For this test, the three-phase currents and voltage waveforms are shown in Fig. 12a and b, respectively. Figure 12c presents the cross-coherence coefficients (*Ci*_*ab*_*, Ci*_*bc*_*,* and *Ci*_*ca*_) of the three-phase current signals, and Fig. 12d introduces the cross-coherence coefficients (*Cv*_*ab*_*, Cv*_*bc*_*,* and *Cv*_*ca*_) of the three-phase voltage signals. Figure 13a plots the auto-coherence coefficients (*Ci*_*a*_*, Ci*_*b*_*,* and *Ci*_*c*_) of each phase current signal, and Fig. 13b shows the auto-coherence coefficients (*Cv*_*a*_*, Cv*_*b*_*,* and *Cv*_*c*_) of each phase voltage signal. Figure 13c depicts the cross-coherence coefficients (*Cvi*_*a*_*, Cvi*_*b*_*,* and *Cvi*_*c*_) estimated between the phase voltage and current signals. The fifteen coherence coefficients calculated for the three voltages and current are determined at each data window. According to the conditions listed in Tables 1 and 2, the coherence technique is able to detect the voltage and current unbalance and disturbance in the power grid. It is also obvious from Figs. 12c, d, 13a, b and c that when the series fault *(A open)* occur, the three coherence coefficients in each figure are unequal and unstable during the fault time, while their values are settled at a fixed value after the fault clearance. Consequently, the protective relay sends a tripping order to isolate the protected generator, as shown in Fig. 13d. In this test, the red flags confirm the states of the current unbalance, voltage unbalance, PF disturbance, current disturbance, and voltage disturbance.

### Test 6: Series fault (C open)

**Fig. 14:**
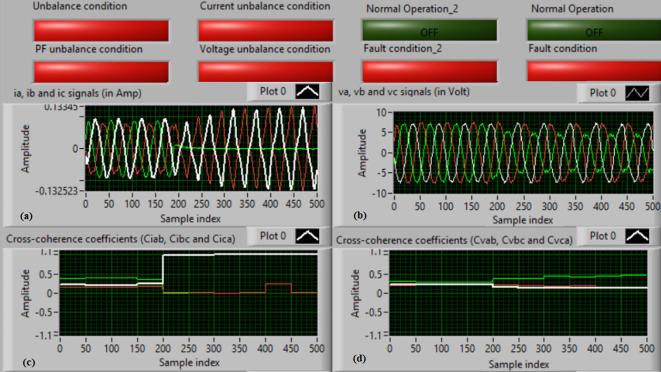
Results for experimental test 6. (**a**) Three-phase current signals* (i*_*a*_*, i*_*b*_ and *i*_*c*_*),* (**b**) Three-phase voltage signals* (v*_*a*_*, v*_*b*_ and *v*_*c*_*),* (**c**) The cross-coherence coefficients (*Ci*_*ab*_*, Ci*_*bc*_ and *Ci*_*ca*_), and (**d**) The cross-coherence coefficients (*Cv*_*ab*_*, Cv*_*bc*_*,* and *Cv*_*ca*_).

**Fig. 15:**
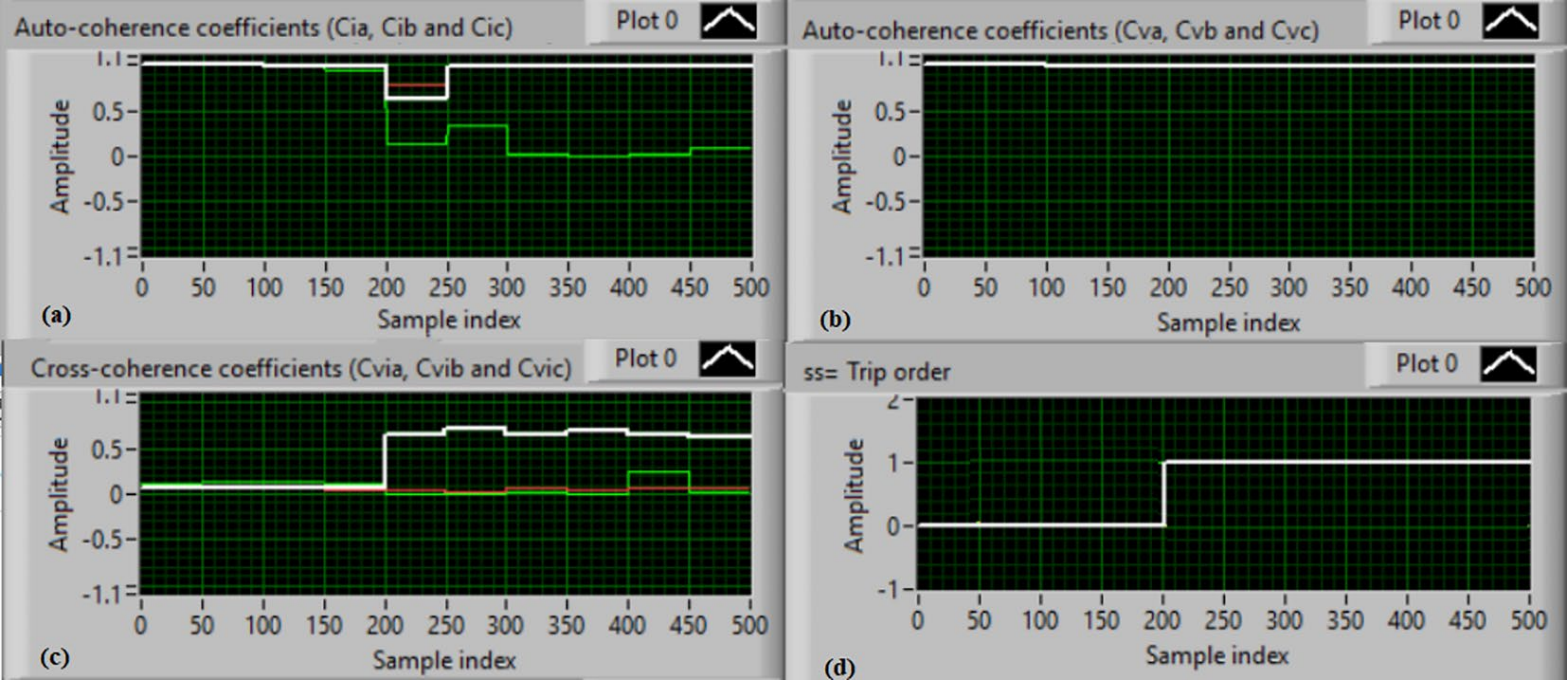
Results for experimental test 6 (Continued). (**a**) The auto-coherence coefficients (*Ci*_*a*_*, Ci*_*b*_ and *Ci*_*c*_), (**b**) The auto-coherence coefficients (*Cv*_*a*_*, Cv*_*b*_*,* and *Cv*_*c*_), (**c**) The cross-coherence coefficients (*Cvi*_*a*_*, Cvi*_*b*_*,* and *Cvi*_*c*_), and (**d**) The relay response.

For this experimental test, the three-phase currents and voltage signals are depicted in Fig. 14a and b, respectively. Figure 14c offers the cross-coherence coefficients (*Ci*_*ab*_*, Ci*_*bc*_*,* and *Ci*_*ca*_) of the three-phase current signals, and Fig. 14d shows the cross-coherence coefficients (*Cv*_*ab*_*, Cv*_*bc*_*,* and *Cv*_*ca*_) of the three-phase voltage signals. Figure 15a presents the auto-coherence coefficients (*Ci*_*a*_*, Ci*_*b*_*,* and *Ci*_*c*_) of each phase current signal, and Fig. 15b displays the auto-coherence coefficients (*Cv*_*a*_*, Cv*_*b*_*,* and *Cv*_*c*_) of each phase voltage signal. Figure 15c introduces the cross-coherence coefficients (*Cvi*_*a*_*, Cvi*_*b*_*,* and *Cvi*_*c*_) computed between the phase voltage and current signals. The fifteen coherence coefficients computed for the three voltages and current are obtained at each data window. According to the rules noted in Tables 1 and 2, the coherence method has the ability to recognize the voltage and current unbalance and disturbance in the power network. It can be clear from Figs. 14c, d, 15a, b and c that when the series fault *(C open)* occur, the three coherence coefficients in each figure are varied and unsymmetrical during the fault interval. Whereas, their values are stationary and stable before the fault inception time. Thus, the proposed protection issues a tripping signal to isolate the protected SG, as given in Fig. 15d. In this test, the red flags assure the situations of the current unbalance, voltage unbalance, PF disturbance, current disturbance, and voltage disturbance.

### Test 7: Series fault (A, B and C open)

**Fig. 16:**
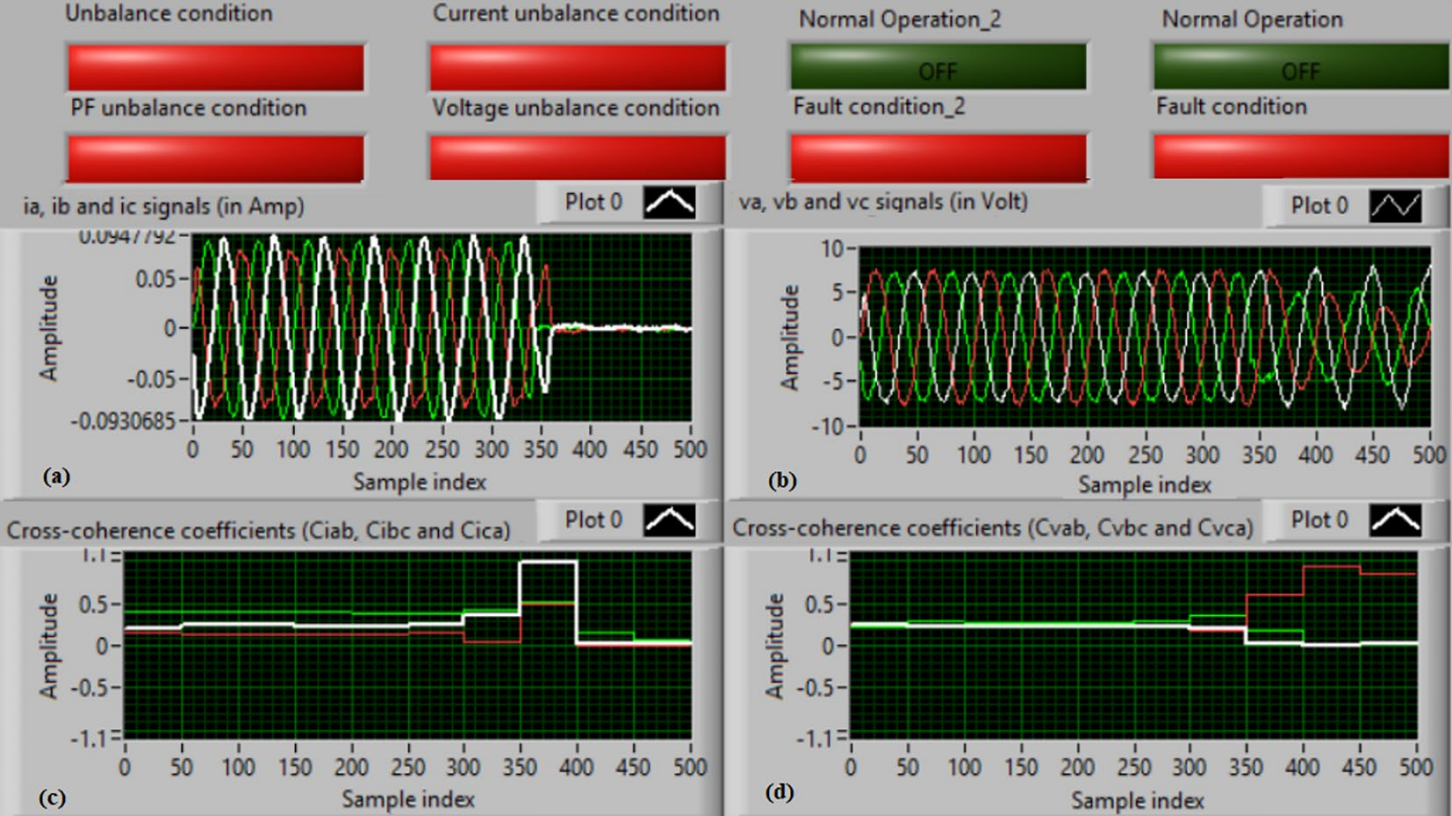
Results for experimental test 7. (**a**) Three-phase current signals* (i*_*a*_*, i*_*b*_ and *i*_*c*_*),* (**b**) Three-phase voltage signals* (v*_*a*_*, v*_*b*_ and *v*_*c*_*),* (**c**) The cross-coherence coefficients (*Ci*_*ab*_*, Ci*_*bc*_ and *Ci*_*ca*_), and (**d**) The cross-coherence coefficients (*Cv*_*ab*_*, Cv*_*bc*_*,* and *Cv*_*ca*_).

**Fig. 17:**
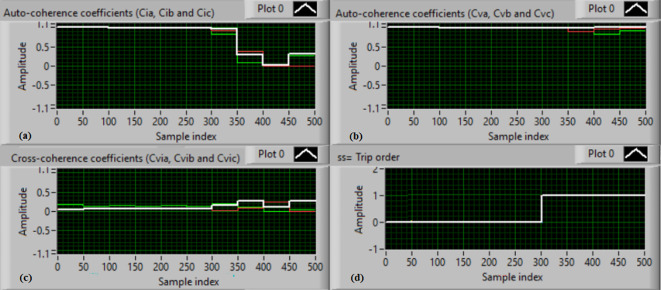
Results for experimental test 7 (Continued). (**a**) The auto-coherence coefficients (*Ci*_*a*_*, Ci*_*b*_ and *Ci*_*c*_), (**b**) The auto-coherence coefficients (*Cv*_*a*_*, Cv*_*b*_*,* and *Cv*_*c*_), (**c**) The cross-coherence coefficients (*Cvi*_*a*_*, Cvi*_*b*_*,* and *Cvi*_*c*_), and (**d**) The relay response.

For this test, the three-phase currents and voltage waveforms are shown in Fig. 16a and b, respectively. Figure 16c presents the cross-coherence coefficients (*Ci*_*ab*_*, Ci*_*bc*_*,* and *Ci*_*ca*_) of the three-phase current signals, and Fig. 16d introduces the cross-coherence coefficients (*Cv*_*ab*_*, Cv*_*bc*_*,* and *Cv*_*ca*_) of the three-phase voltage signals. Figure 17a plots the auto-coherence coefficients (*Ci*_*a*_*, Ci*_*b*_*,* and *Ci*_*c*_) of each phase current signal, and Fig. 17b shows the auto-coherence coefficients (*Cv*_*a*_*, Cv*_*b*_*,* and *Cv*_*c*_) of each phase voltage signal. Figure 17c depicts the cross-coherence coefficients (*Cvi*_*a*_*, Cvi*_*b*_*,* and *Cvi*_*c*_) computed between the phase voltage and current signals. The fifteen coherence coefficients calculated for the three voltages and current are determined at each data window. According to the conditions listed in Tables 1 and 2, the coherence technique is able to detect the voltage and current unbalance and trouble in the power grid. It is also seen from Figs. 16c, d, 17a, b and c that when the series fault *(A, B* and* C open)* exists, the three coherence coefficients in each figure are unequal and unstable during the fault time. Whereas, their values are settled at a fixed value before the fault occurrence. Hence, the protective relay sends a tripping order to isolate the protected generator, as shown in Fig. 17d. In this test, the red flags confirm the occurrence of the current unbalance, voltage unbalance, PF disturbance, current disturbance, and voltage disturbance. According to the experimental results depicted in Figs. 12, 13, 14, 15, 16, 17, it is reasonable to deduce that the proposed protection approach based on the fifteen coherence coefficients can identify the voltage and current unbalance and trouble (originated due to the series faults) in the three-phase power system speedily and precisely.

### Quantitative findings: Imbalance and disturbance indicators

Table 5 includes the quantitative findings of the five imbalance and disturbance indicators (*Uv*,* Ui, Dvi*_*,*_* Dv* and* Di*) during the fault conditions for each case study.

**Table 5:** The quantitative findings of the imbalance and disturbance indicators.

Case number	Quantitative findings during the fault conditions					
Case 1	Coherence value corresponding to MAE	*Ci*_*sx*_	*Cv*_*sx*_	*Cvi*_*s*_	*Ci*_*s*_	*Cv*_*s*_
		+ 0.95	0.8	0.0	0.0	0.0
	Imbalance and disturbance indicators	*Ui*	*Uv*	*Dvi*	*Di*	*Dv*
		+ 0.70	+ 0.55	+ 1.0	+ 1.0	+ 1.0
Case 2	Coherence value corresponding to MAE	*Ci*_*sx*_	*Cv*_*sx*_	*Cvi*_*s*_	*Ci*_*s*_	*Cv*_*s*_
		+ 0.90	+ 1.0	0.0	0.0	0.0
	Imbalance and disturbance indicators	*Ui*	*Uv*	*Dvi*	*Di*	*Dv*
		+ 0.65	+ 0.75	+ 1.0	+ 1.0	+ 1.0
Case 3	Coherence value corresponding to MAE	*Ci*_*sx*_	*Cv*_*sx*_	*Cvi*_*s*_	*Ci*_*s*_	*Cv*_*s*_
		+ 0.70	+ 1.0	0.0	0.0	0.0
	Imbalance and disturbance indicators	*Ui*	*Uv*	*Dvi*	*Di*	*Dv*
		+ 0.45	+ 0.75	+ 1.0	+ 1.0	+ 1.0
Case 4	Coherence value corresponding to MAE	*Ci*_*sx*_	*Cv*_*sx*_	*Cvi*_*s*_	*Ci*_*s*_	*Cv*_*s*_
		+ 0.90	+ 1.0	0.0	0.0	0.0
	Imbalance and disturbance indicators	*Ui*	*Uv*	*Dvi*	*Di*	*Dv*
		+ 0.65	+ 0.75	+ 1.0	+ 1.0	+ 1.0
Case 5	Coherence value corresponding to MAE	*Ci*_*sx*_	*Cv*_*sx*_	*Cvi*_*s*_	*Ci*_*s*_	*Cv*_*s*_
		+ 1.0	+ 0.55	0.0	+ 0.1	+ 0.9
	Imbalance and disturbance indicators	*Ui*	*Uv*	*Dvi*	*Di*	*Dv*
		+ 0.75	+ 0.30	+ 1.0	+ 0.9	+ 0.1
Case 6	Coherence value corresponding to MAE	*Ci*_*sx*_	*Cv*_*sx*_	*Cvi*_*s*_	*Ci*_*s*_	*Cv*_*s*_
		+ 1.0	+ 0.50	0.0	0.0	+ 0.95
	Imbalance and disturbance indicators	*Ui*	*Uv*	*Dvi*	*Di*	*Dv*
		+ 0.75	+ 0.25	+ 1.0	+ 1.0	+ 0.05
Case 7	Coherence value corresponding to MAE	*Ci*_*sx*_	*Cv*_*sx*_	*Cvi*_*s*_	*Ci*_*s*_	*Cv*_*s*_
		+ 1.0	+ 0.90	0.0	0.0	+ 0.8
	Imbalance and disturbance indicators	*Ui*	*Uv*	*Dvi*	*Di*	*Dv*
		+ 0.75	+ 0.65	+ 1.0	+ 1.0	+ 0.2

## Overall technique evaluation

### Protection accuracy and reliability assessment

The proposed algorithm’s performance was monitored over a period of two months. It was found that the algorithm operated 230 times, and 228 of those trips were correct. Two tripping decisions were not issued, and the protection was restrained 40 times without any mal-function in all cases of normal operating conditions. Table 6 shows the percentages of accuracy, dependability, security, and reliability for the proposed protection scheme under both faults and measurement errors^43^.

**Table 6:** Performance’s evaluation for the proposed protection scheme.

Power model situation	Number of experiments	Mal-function times number
Fault situation	230	2
Normal operating situation	40	0
Total number	270	2
Technique performance’s evaluation		
The percentages of accuracy, dependability, security, and reliability for the proposed protection scheme	T_1_ = Total number of experiments	270
	T_2_ = Total number of trips	230
	T_3_ = Number of correct trips	228
	T_4_ = The times number of the protective relay failed to issue trip decision	2
	T_5_ = Number of desired trips	228 + 2 = 230
	T_6_ = Number of incorrect trips	230 – 228 = 2
	T_7_ = Number of desired trips + Number of incorrect trips	230 + 2 = 232
	$$Accuracy = \begin{array}{*{20}c} {} \\ {} \\ \end{array} \frac{{T_{1} \begin{array}{*{20}c} {} \\ {} \\ \end{array} - \begin{array}{*{20}c} {} \\ {} \\ \end{array} T_{4} \begin{array}{*{20}c} {} \\ {} \\ \end{array} - \begin{array}{*{20}c} {} \\ {} \\ \end{array} T_{6} }}{{T_{1} }}\begin{array}{*{20}c} {} \\ {} \\ \end{array} \times 100\begin{array}{*{20}c} {} \\ {} \\ \end{array} (\% )$$	(270 – 2 – 2)/270 × 100 = (266/270) × 100 = 98.52%
	$${\text{Sec}} urity = \begin{array}{*{20}c} {} \\ {} \\ \end{array} \frac{{T_{3} }}{{T_{2} }}\begin{array}{*{20}c} {} \\ {} \\ \end{array} \times 100\begin{array}{*{20}c} {} \\ {} \\ \end{array} (\% )$$	(228/230) × 100 = 99.13%
	$$Dependability = \begin{array}{*{20}c} {} \\ {} \\ \end{array} \frac{{T_{3} }}{{T_{5} }}\begin{array}{*{20}c} {} \\ {} \\ \end{array} \times 100\begin{array}{*{20}c} {} \\ {} \\ \end{array} (\% )$$	(228/230) × 100 = 99.13%
	$${\text{Re}} liability = \begin{array}{*{20}c} {} \\ {} \\ \end{array} \frac{{T_{3} }}{{T_{7} }}\begin{array}{*{20}c} {} \\ {} \\ \end{array} \times 100\begin{array}{*{20}c} {} \\ {} \\ \end{array} (\% )$$	228/(230 + 2) × 100 = 98.28%
The protection operating time	The relay speed is determined by the total time required for data transmission and processing to detect the fault and unbalance events. Therefore, it relies on the pre-determined data set quantity (*N*)	The operating time = 20 ms

### Protection technique properties

The proposed digital protection has the following significant features:

1. It is able to function online for protecting various elements in different arrangements of power networks, such as conventional and smart grids, and it is applicable in large-scale power grids with different voltage ratings,
2. It can only use two kinds of mathematical equations for computing nine cross-coherence coefficients and six auto-coherence coefficients (i.e., fifteen coherence coefficients) of the three-phase voltage and current measurements,
3. A new proposal for tripping-characteristics based on the coherence indices computed for the three-phase voltages and currents is presented. The proposal does not react during the balanced and normal operating conditions, while it active in the event of the power system contingency,
4. It is capable of recognizing the situations of the imbalance/disturbance for the three-phase voltages and currents. In other words, it can respond to abnormal events such as shunt fault, series fault, unbalance voltage, unbalance current, loss-of-synchronism, underfrequency, overfrequency, undervoltage, and overvoltage,
5. It is characterized by a high level of functionality integration since the unbalance and fault digital detectors can be implemented using the same numerical method,
6. It can only use one predetermined threshold value for the coherence coefficients to find out if there is an imbalance or fault situation,
7. The numerical values of the three-phase voltages and currents, as well as the relay settings that include the data set area and the coherence deviations, are sufficient for processing the suggested protection algorithm,
8. It is based on a simple computational technique that allows for easy adjustment of the relay setting,
9. The coherence coefficients can value accurately the intensity level of the disturbance/unbalance for both voltage and current waveforms,
10. It is capable of accurately assessing the system power factor,
11. It can find the disturbance/unbalance situations quickly and precisely using the coherence indices computed for the three-phase voltage and current measurements,
12. It is advantageous because it is simple, functional, robust, swift, secure, dependable, reliable and accurate,
13. The protection speed, sensitivity and security can be controlled using the change in the data set quantity and the predetermined deviation settings of the coherence estimators,
14. It can do the detection and assessment functions simultaneously for the fault/unbalance events (i.e., the coherence coefficient is a measure of the association between any two signals/data sets, and reveals the degree of synchrony between them). Therefore, it is considered a suitable estimator for evaluating the association degree, and identifying sudden changes between the two signals/data sets at the same time,
15. It is possible to work without a low-pass filter since the moving data window is considered a digital filter. This clears the decent ripples, temporary faults and DC offset components of the electrical measurements,
16. The specifications of the protected elements of the power system or measurement transformers (i.e., PTs and CTs) will not affect the technique performance as long as the system and the transformers are well designed.
17. The proposed algorithm enhances a protection dependability, availability and operational flexibility, resulting in an improved relay performance. Furthermore, it provides redundant protection functions for the following reasons:

- The protection system uses five sub-algorithms to maintain a correct and normal operation of the protection system if one critical sub-algorithm is not operating. Thus, the redundant sub-algorithms improve system reliability by maintaining both dependability and security.
- The backup protection maintains the dependability of the total protection system during incorrect operation of the primary protection. The backup function is not part of the primary protection for a certain segment of the power system, and maintains dependability at the expense of security.
- The protection system is available when all functions necessary (to isolate a fault for a specific zone of protection within the desired operating time) are operating normally. Redundancy therefore increases reliability by ensuring the protection system is available to protect a specific part of the system.
- The protection system provides a complete snd redundant protection for short-circuit current and voltage-based protection functions.
- A combination of three sub-algorithms based on nine cross-coherence coefficients and two sub-algorithms based on six auto-coherence coefficients is needed to properly execute the protection functions for detecting unbalance and fault events.

### Critical comparison

Table 7 presents a comparison between the currently submitted work and the previously published work ^38^ in the literature.

**Table 7:** Critical comparison between the proposed method and the previously published method ^43^.

Sr. N.	Item	The published work^43^	The current work
1	Mathematical formulas	Its algorithm used the mathematical formulas of the alienation coefficients derived utilizing the MSC coefficients computed for the three-phase voltages and currents. Where, *A* = *1 – MSC*	Its algorithm applies directly the mathematical equations of the MSC coefficients calculated for the three-phase voltages and currents
2	Tripping-characteristic curves	The tripping-characteristic curves were dependent on the alienation coefficients. Each curve is restricted between the two values of 0.0 and + 1.0	New tripping-characteristic curves are based on the MSC coefficients. Each curve ranges between the two values of 0.0 and + 1.0
3	The algorithm response speed	The response speed of the alienation-based algorithm was slower	The response speed of the MSC-based algorithm is faster than the alienation-based algorithm
4	Di-symmetry and disturbance indices	The di-symmetry and disturbance indices were measured using the alienation coefficients	The di-symmetry and disturbance indices are valued using the MSC coefficients. Furthermore, five new indices (*U*_*v*_*, U*_*i*_*, D*_*vi*_*, D*_*v*_ and* D*_*i*_) for evaluating the intensity levels of disturbance and di-symmetry for the three-phase voltages and currents taken at the terminals of SG stator windings. In this proposal, the Maximum Absolute Error (MAE) concept of the MSC estimators is used to obtain two di-symmetry indicators (*U*_*v*_ and *U*_*i*_) and three disturbance indicators (*D*_*vi*_*, D*_*v*_* and D*_*i*_)
5	Performance assessment	The accuracy, security, dependability, and reliability percentages of the previous protection algorithm were the lowest Accuracy = 95.83% Security = 96.47% Dependability = 97.62% Reliability 94.25%	The accuracy, security, dependability, and reliability percentages of the current protection algorithm are the largest Accuracy = 98.52% Security = 99.13% Dependability = 99.13% Reliability = 98.28%
6	Protection sensitivity	The relay sensitivity is related to the selected numerical values of alienation setting deviations. The relay will be more sensitive as the selected setting values are lower ${\text{Sensitivity factor}} \alpha \left| {\frac{1}{{\text{Alienation setting deviation}}}} \right|$ In the event of low fault current, the sensitivity can be improved by decreasing both the data window size and the restraining zones located inside the proposed characteristics based on alienation coefficients	The relay sensitivity is related to the selected numerical values of MSC setting deviations. The relay will be more sensitive as the selected setting values are lower ${\text{Sensitivity factor}} \alpha \left| {\frac{1}{{\text{MSC setting deviation}}}} \right|$ In the event of low fault current, the sensitivity can be improved by decreasing both the data window size and the restraining zones located inside the proposed characteristics based on MSC coefficients
7	Protection security	In the event of overload current and CT linear errors, the security can be enhanced by increasing both the data window size and the restraining zones located inside the proposed characteristics based on alienation coefficients,	In the event of overload current and CT linear errors, the security can be enhanced by increasing both the data window size and the restraining zones located inside the proposed characteristics based on MSC coefficients,
8	The control to the algorithm processing time	For both methods, the algorithm processing time is controllable using the quantity of data window,	
9	Multi-functions	Both approaches can run various protection functions for detecting and evaluating the disturbance and imbalance of three-phase voltage and current waves	

## Prominent contributions

The contributions to knowledge in this article are listed as follows:

1. A new protection method is developed to detect and measure the voltage and current imbalances and disturbances online using the cross-coherence and auto-coherence coefficients calculated for the three-phase voltage and current curves,
2. Five coherence-based sub-algorithms are integrated into a digital protection scheme for detecting and evaluating unbalance and fault conditions,
3. The digital protection system employs only two types of mathematical formulas that are applicable for computing nine cross-coherence indices and six auto-coherence indices of voltage and current waves,
4. A group of novel tripping-curves can be developed based on the coherence coefficients calculated for the voltages and currents per each data set, which can be used to operate the protection algorithm during the fault and unbalance times to keep the system from being damaged. Whereas, it holds the protection operation in the balanced and normal operating situations for the power network,
5. The algorithm can easily figure out the appropriate setting for the coherence estimators to differentiate between the acceptable and unacceptable imbalance. Furthermore, the sensitivity of the protective relay can be controlled,
6. The proposed approach has the superior ability among other existing methods at finding the voltage and current imbalances/disturbances quickly within one cycle time. Moreover, it is able to control the time it takes to find out if the voltages or currents are out of balance by amending the data window quantity, and
7. For finding and assessing the unbalance condition, the coherence coefficients combine the effects of both negative and zero sequence components. Furthermore, the coherence coefficient can sense a change in any power quality parameter of the electrical waveform (such as magnitude, frequency, phase shift or symmetry). Thus, the coherence statistic is considered to be a proper measure for evaluating the di-symmetry factor.

## Conclusions

In this work, a novel algorithm has been presented to monitor and assess the imbalances/disturbances of the voltage and current signals in the three-phase synchronous machines. The proposed technique is considered to be a backup protection for the SG stator windings, which trips appropriate CBs only if the main protections fail to detect the fault presence or miss opening them. The technique has used the three-phase voltage and current measurements of the SG stator windings, and it has computed the fifteen coherence coefficients for the six electrical signals in order to identify the system situation precisely and quickly. Various protection functions (such as voltage and current fault detectors, voltage and current unbalance detectors and power factor measurements) are integrated into one protection system based on the fifteen coherence coefficients. A practical power model has been constructed, and several experimental tests have been accomplished to value the effectiveness of the proposed protection scheme. The experimental results have demonstrated a high quality of the proposed protection for detecting the unbalanced/disturbed voltages and currents, which its operating time is within one cycle. The results of the extensive experimental tests have shown that the relay accuracy is approximately 98.52%, and the protection dependability and security are found to be nearly 99.13%, resulting in the relay reliability is roughly 98.28%. Moreover, the detection technique using the coherence method is a simple, smart, dynamic, potent and functional protection setup. Besides, the algorithm can run online at different speeds. Furthermore, the coherence settings do not require any offline studies, so they can be modified easily. The method is suitable for any synchronous machine or power system with different specifications.

## Electronic supplementary material

Below is the link to the electronic supplementary material.

Supplementary Material 1

## Data Availability

All data generated or analysed during this study are included in this published article [and its supplementary information files].

## Abbreviations

*v*_*a*_*(n)*, *v*_*b*_*(n)* and *v*_*c*_*(n)*: The three-phase voltage signals at sample ‘*n*’ measured at the SG terminals of phases *A*, *B* and *C*, respectively
*i*_*a*_*(n)*, *i*_*b*_*(n)* and *i*_*c*_*(n)*: The three-phase current signals at sample ‘*n*’ measured at the SG terminals, of phases *A*, *B* and *C*, respectively
*Cg*_*sx*_*(k)*: The cross-coherence coefficient, on a given frequency (*k*), calculated between each two corresponding data sets of the two electrical signals *(g*_*s*_*(n)* and *g*_*x*_*(n))* for the two phases ‘*S*’ and ‘*X*’, respectively
*Cv*_*sx*_*(k)*: The cross-coherence coefficient, on a given frequency (*k*), calculated between each two corresponding data sets of the two voltage signals *(v*_*s*_*(n)* and *v*_*x*_*(n))* for the two phases ‘*S*’ and ‘*X*’, respectively
*Cv*_*ab*_*(k)*: The cross-coherence coefficient, on a given frequency (*k*), calculated between each two corresponding data sets of the two voltage signals *(v*_*a*_*(n)* and *v*_*b*_*(n))*
*Cv*_*bc*_*(k)*: The cross-coherence coefficient, on a given frequency (k), calculated between each two corresponding data sets of the two voltage signals *(v*_*b*_*(n)* and *v*_*c*_*(n))*
*Cv*_*ca*_*(k)*: The cross-coherence coefficient, on a given frequency (*k*), calculated between each two corresponding data sets of the two voltage signals *(v*_*c*_*(n)* and *v*_*a*_*(n))*
*Ci*_*sx*_*(k)*: The cross-coherence coefficient, on a given frequency (*k*), calculated between each two corresponding data sets of the two current signals *(i*_*s*_*(n)* and *i*_*x*_*(n))* for the two phases ‘*S*’ and ‘*X*’, respectively
*Ci*_*ab*_*(k)*: The cross-coherence coefficient, on a given frequency (*k*), calculated between each two corresponding data sets of the two current signals *(i*_*a*_*(n)* and *i*_*b*_*(n))*
*Ci*_*bc*_*(k)*: The cross-coherence coefficient, on a given frequency (*k*), calculated between each two corresponding data sets of the two current signals *(i*_*b*_*(n)* and *i*_*c*_*(n))*
*Ci*_*ca*_*(k)*: The cross-coherence coefficient, on a given frequency (*k*), calculated between each two corresponding data sets of the two current signals *(i*_*c*_*(n)* and *i*_*a*_*(n))*
*Cgf*_*s*_*(k)*: The cross-coherence coefficient, on a given frequency (*k*), calculated between the phase voltage and current signals *(g*_*s*_*(n)* and *f*_*s*_*(n))* for the phase ‘*S*’
*Cvi*_*a*_*(k)*: The cross-coherence coefficient, on a given frequency (*k*), calculated between each two corresponding data sets of the voltage and current signals *(v*_*a*_*(n)* and *i*_*a*_*(n))*
*Cvi*_*b*_*(k)*: The cross-coherence coefficient, on a given frequency (*k*), calculated between each two corresponding data sets of the voltage and current signals *(v*_*b*_*(n)* and *i*_*b*_*(n))*
*Cvi*_*c*_*(k)*: The cross-coherence coefficient, on a given frequency (*k*), calculated between each two corresponding data sets of the voltage and current signals *(v*_*c*_*(n)* and *i*_*c*_*(n))*
*Cg*_*s*_*(k)*: The auto-coherence coefficient, on a given frequency (*k*), calculated between each two successive data sets shifted from each other by one-cycle interval of the electrical signal *(g*_*s*_*(n)* and *g*_*s*_*(n - N*_*s*_*))* of the ‘*S*’ phase
*Ci*_*s*_*(k)*: The auto-coherence coefficient, on a given frequency (*k*), calculated between each two successive data sets shifted from each other by one-cycle interval of the current signal *(i*_*s*_*(n)* and *i*_*s*_*(n - N*_*s*_*))* of the ‘*S*’ phase
*Ci*_*a*_*(k)*: The auto-coherence coefficient, on a given frequency (*k*), calculated between each two successive data sets shifted from each other by one-cycle interval of the current signal *(i*_*a*_*(n)* and *i*_*a*_*(n - N*_*s*_*))* of the ‘*A*’ phase
*Ci*_*b*_*(k)*: The auto-coherence coefficient, on a given frequency (*k*), calculated between each two successive data sets shifted from each other by one-cycle interval of the current signal *(i*_*b*_*(n)* and *i*_*b*_*(n - N*_*s*_*))* of the ‘*B*’ phase
*Ci*_*c*_*(k)*: The auto-coherence coefficient, on a given frequency (*k*), calculated between each two successive data sets shifted from each other by one-cycle interval of the current signal *(i*_*c*_*(n)* and *i*_*c*_*(n - N*_*s*_*))* of the ‘*C*’ phase
*Cv*_*s*_*(k)*: The auto-coherence coefficient, on a given frequency (*k*), calculated between each two successive data sets shifted from each other by one-cycle interval of the voltage signal *(v*_*s*_*(n)* and *v*_*s*_*(n - N*_*s*_*))* of the ‘*S*’ phase
*Cv*_*a*_*(k)*: The auto-coherence coefficient, on a given frequency (*k*), calculated between each two successive data sets shifted from each other by one-cycle interval of the voltage signal *(v*_*a*_*(n)* and *v*_*a*_*(n - N*_*s*_*))* of the ‘*A*’ phase
*Cv*_*b*_*(k)*: The auto-coherence coefficient, on a given frequency (*k*), calculated between each two successive data sets shifted from each other by one-cycle interval of the voltage signal *(v*_*b*_*(n)* and *v*_*b*_*(n - N*_*s*_*))* of the ‘*B*’ phase
*Cv*_*c*_*(k)*: The auto-coherence coefficient, on a given frequency (*k*), calculated between each two successive data sets shifted from each other by one-cycle interval of the voltage signal *(v*_*c*_*(n)* and *v*_*c*_*(n - N*_*s*_*))* of the ‘*C*’ phase
*MSC*: Mean Squared Coherence
*DFT*: Discrete Fourier Transform
*RMS*: The Root Mean Square
*Δ*_*1*_, *Δ*_*2*_ and *Δ*_*3*_: The cross-coherence setting deviations; they lie within the values range of 0.0 and 0.25
*Δ*_*4*_ and *Δ*_*5*_: The auto-coherence setting deviations; they lie within the values range of 0.0 and 0.25
*S*, *X* and *Y*: The phase designation *A*, *B* or *C*; but they are not the same phase
*v*_*s*_*(n)*: The voltage numerical value at sample index *n* for phase ‘*S*’
*v*_*x*_*(n)*: The voltage numerical value at sample index *n* for phase ‘*X*’
*i*_*s*_*(n)*: The current numerical value at sample index *n* for phase ‘*S*’
*i*_*x*_*(n)*: The current numerical value at sample index *n* for phase ‘*X*’
*v*_*s*_* (n-N*_*s*_*)*: The numerical value of the phase voltage signal *(v*_*s*_*)* at sample index one-cycle prior to *n* for ‘*S* phase’
*i*_*s*_* (n-N*_*s*_*)*: The numerical value of the phase current signal *(i*_*s*_*)* at sample index one-cycle prior to n for ‘*S* phase’
*n*: The sample index/order (in the time domain)
*k*: The *k*'th frequency component
*N*_*s*_: The number of samples per cycle used in the simulation, (*N*_*s*_ = 50 samples/cycle)
*N*: The number of samples per data set used in the algorithm (*N* ≤ *Ns*_)_
*T*_*c*_: The periodic cycle, (*T*_*c*_ = 20 *mSec*)
*F*_*c*_: The fundamental frequency of one periodic cycle, (*F*_*c*_ = 50 Hz)
*T*_*s*_: The sampling time interval, (*T*_*s*_ = 0.4 *mSec*)
*F*_*s*_: The sampling frequency, (*F*_*s*_ = 2.5 kHz)
*AE* and *MAE*: The Absolute Error and the Maximum Absolute Error
*Uv* and *Ui*: The imbalance indicators for the coherence estimators of the three-phase voltages and currents, respectively
*Dvi*: The disturbance indicator for the coherence estimators of both single-phase voltage and current
*Dv* and *Di*: The disturbance indicators for the coherence estimators of the single-phase voltage and current, respectively
*ω*: The angular velocity of the power system (ω = 2 πf)
*R*_*f*_: The fault resistance imposed from the faulted point at the terminals of the SG stator windings to the ground point in the case of the ground fault, or inserted between the two faulted phases in case of the phase fault
*t*_*f*_: The fault inception time
*N*_*f*_: The sample index at which the fault occurs
*N*_*t*_: The full number of samples per the display time
*SLNF*: Single line-to-neutral fault
*DLNF*: Double line-to-neutral fault
*DLF*: Double line fault
*3LNF*: Three line-to-neutral fault
*SG*: Synchronous Generator
*R*_*n*_: Generator grounding resistance through the neutral point
*V*_*n*_: The nominal voltage of the synchronous generator (SG)
*I*_*n*_: The nominal current of the synchronous generator (SG)
*SLD*: Single Line Diagram
*PF*: Power Factor
*FD*: Fault Detection
*FL*: Fault Location
*VT*: Voltage Transformer
*CT*: Current Transformer
*VTR*: Voltage Transformer Ratio
*CTR*: Current Transformer Ratio
*R*_*b*_: The current transformer (CT) burden (it is 1 Ω)
*R*_*CT*_: The current transformer (CT) secondary winding resistance
*R*_*lead*_: The lead resistance connected between the current transformer (CT) terminals and the burden
*CB*: Circuit Breaker
