# Supplementary material for: Experimental performance verification of an intelligent detection and assessment scheme for disturbances and imbalances of three-phase synchronous machine output using coherence estimators
Source: Sci Rep. 2024 Nov 1;14:26278. doi: 10.1038/s41598-024-76343-8 (PMC11530546; doi:10.1038/s41598-024-76343-8)
Supplement: Supplementary file 1 — Supplementary Material 1 [file 41598_2024_76343_MOESM1_ESM.doc]

**Appendix 1:** Parameters’ data of the components of the power system model

| **The parameters of the system components** | **Data** |
| --- | --- |
| **Three-phase synchronous generator (SG):**  Type  Rated power  Rated speed  Rated line voltage  Rated line current  Rated frequency  Number of pair poles  Excitation voltage  Excitation current  Power factor  Neutral grounding impedance (*R_n_*) | *STC-8 kW (Star connection)*  *10 kVA*  *1500 rpm*  *380 V*  *15.1 A*  *50 Hz*  *2*  *100 V*  *4 A*  *0.8*  *Isolated* |
| **Single-phase induction motor (Prime mover):**  Type  Rated power  Nominal phase voltage  Rated current  Rated speed  Nominal frequency  Nominal power factor | *(Capacitor start motor)*  *YC112L2-4*  *5.5 Hp (4 kW)*  *220 V*  *28 A*  *1400 rpm*  *50 Hz*  *0.80* |
| **Three-phase power transformer:**  Rated power  Rated line voltage  Number of Tapes  Rated line current  Neutral grounding impedance | *(Star connection)*  *5 kVA*  *400 V*  *20 Tapes*  *7.5 A*  *Isolated* |
| **Three-phase induction motor (Load):**  Rated power  Rated line voltage  Rated line current  Rated speed  Nominal frequency  Operating power factor | *4 kW (Star connection)*  *400 V*  *6.42 A*  *1440 rpm*  *50 Hz*  *0.79* |
| **Current transformers (CTs):**  CTR  Frequency  CT class  Rated burden  CT burden | *300/5*  *47…50…63 Hz*  *1.0*  *2.5 VA*  *1 Ω* |
| **Voltage Transformers (VTs)**  VTR  VT class  Nominal frequency  Rated burden | *220 / 5*  *0.5*  *50 Hz*  *5 VA* |
| **Miniature Circuit Breaker (MCB1)**  Phase type  Rated current  Rated voltage | Single phase  63 A  380 V |
| **Miniature Circuit Breaker (MCB2)**  Phase type  Rated current  Rated voltage | Three phase  20 A  380 V |
| **Miniature Circuit Breakers (MCB3 MCB4 and MCB5)**  Phase type  Rated current  Rated voltage | Single phase  20 A  380 V |
